# Identifying UK travellers at increased risk of developing pneumococcal infection: a novel algorithm

**DOI:** 10.1093/jtm/taab063

**Published:** 2021-05-12

**Authors:** Gillian Ellsbury, James Campling, Harish Madhava, Mary Slack

**Affiliations:** 1 Vaccines Medical Affairs, Pfizer Ltd, Tadworth, KT20 7NS, UK; 2 School of Medicine & Dentistry, Gfiffith University Gold Coast campus, Queensland 4222, Australia

**Keywords:** Travel, vaccination, traveller, risk algorithm, pneumococcal disease, prevention

## Abstract

**Background:**

In 2016, the travel subcommittee of the UK Joint Committee on Vaccination and Immunisation (JCVI) recommended that 13-valent PCV (PCV13) could be offered to travellers aged over 65 years, visiting countries without infant PCV immunization programmes. This study aimed to identify, collate and review the available evidence to identify specific countries where UK travellers might be at an increased risk of developing pneumococcal infection. The data were then used to develop an algorithm, which could be used to facilitate implementation of the JCVI recommendation.

**Methods:**

We conducted a systematic search of the published data available for pneumococcal disease, PCV vaccine implementation, coverage data and programme duration by country. The primary data sources used were World Health Organization databases and the International Vaccine Access Centre Vaccine Information and Epidemiology Window-hub database. Based on the algorithm, the countries were classified into ‘high overall risk’, ‘intermediate overall risk’ and ‘low overall risk’ from an adult traveller perspective. This could determine whether PCV13 should be recommended for UK adult travellers.

**Results:**

A data search for a total of 228 countries was performed, with risk scores calculated for 188 countries. Overall, 45 countries were classified as ‘high overall risk’, 86 countries as ‘intermediate overall risk’, 57 countries as ‘low overall risk’ and 40 countries as ‘unknown’.

**Conclusion:**

To our knowledge this is the first attempt to categorize the risk to UK adult travellers of contracting pneumococcal infection in each country, globally. These findings could be used by national travel advisory bodies and providers of travel vaccines to identify travellers at increased risk of pneumococcal infection, who could be offered PCV immunization.

## Introduction

*Streptococcus pneumoniae* (pneumococcus) is a Gram-positive diplococcus, which is a major cause of morbidity and mortality in children and the elderly worldwide.^1^^,^^2^ Pneumococci are classified into >96 serotypes, based on their polysaccharide capsule. Diseases caused by pneumococci include serious infections, such as meningitis, bacteraemia and pneumonia.^1^^,^^2^ Young children (<2 years), older adults (≥65 years) and those with certain underlying comorbidities (e.g. diabetes, heart or respiratory disease) or who are immunocompromised are at increased risk of pneumococcal infection.^1–3^

Two types of pneumococcal vaccine are currently available. These are the plain polysaccharide vaccine (PPSV23)^4^ and the pneumococcal conjugate vaccines (PCV10 and PCV13).^5^^,^^6^ Higher valency conjugate vaccines are also in development. PPSV23 is licensed for all ages over 2 years and contains polysaccharides of serotypes 1, 2, 3, 4, 5, 6B, 7F, 8, 9N, 9V, 10A, 11A, 12F, 14, 15B, 17F, 18C, 19A, 19F, 20, 22F, 23F and 33F.^7^ PCV10 is licensed for infants aged between 6 weeks and 5 years and PCV13 is licensed from 6 weeks of age, containing polysaccharides for 10 and 13 serotypes, respectively.^5^^,^^6^ PCVs offer several advantages over PPSV23: PCVs elicit a T-cell dependent response and can be administered to infants from 6 weeks of age; PCVs induce immunological memory and prevent nasopharyngeal carriage acquisition of vaccine serotypes (VTs) and, therefore, produce herd protection in unvaccinated individuals^1^; and PCVs generate a higher immune response in older adults preventing both bacteraemic and non-bacteraemic pneumonia caused by VTs.^7^

PPSV23 has moderate effectiveness in preventing invasive pneumococcal disease (IPD) among the elderly, but its effectiveness against IPD in high-risk adults is lower.^8–10^ Effectiveness data for protection against non-IPD by PPSV23 has been inconsistent.^11^ In addition, the vaccine does not provide protection against nasopharyngeal carriage thereby preventing the development of herd immunity.^8^ Furthermore, PPSV23 does not induce immunological memory.^12^

The introduction of PCV infant vaccination programmes has decreased the overall incidence of pneumococcal disease in children through direct protection, and in other age groups through indirect protection.^2^^,^^13^^,^^14^ Despite introduction of these vaccination programmes and global public health efforts, in 2019, the World Health Organization (WHO) estimated that only 48% of infants globally were receiving the full dose of a pneumococcal vaccine, as per individual country recommendations and national immunization programme.^15^ Many older adults from the UK travel abroad, increasing their risk of exposure to potential pathogens, including pneumococcal VTs, which may not be prevalent in the UK.

In the UK, the Joint Committee on Vaccination and Immunisation (JCVI) provides independent advice to the Department of Health on immunization. In May 2016, the JCVI reviewed whether PCV13 specifically should be offered to travellers aged over 65 years in circumstances where countries did not have an established infant pneumococcal programme.^16^ The JCVI travel subcommittee advised that PCV13 could be offered to travellers aged over 65 years with the following specific travel conditions: staying longer than a month, working with local communities, or in close contact with the local population, in countries without established programmes.^16^ The JCVI travel subcommittee recognized that the incidence of travel related pneumococcal disease was unknown, and travel history is not routinely collected on cases. Despite the limitations in availability of travel specific data, the recommendation was made by JCVI to consider PCV13 for those travellers in the groups outlined.^16^ We are unaware of any similar recommendations in other countries. In the UK, PPV23 vaccination is offered to all over 65 years with PCV13 only recommended for adults in high-risk groups.^17^

There is an increased risk of pneumococcal disease in mass gatherings. The Hajj is an annual pilgrimage to Makkah Al-Mukarramah (Mecca) in the Kingdom of Saudi Arabia, with ~2.5 million pilgrims from over 180 countries, in both the northern and southern hemispheres, congregating over 5 days in a confined area on the outskirts of the city of Mecca.^18^ More than a quarter of the Hajjis are aged >60 years and a considerable proportion have underlying comorbidities.^19^^,^^20^ Other religious mass gatherings include the annual Arbeen, Iraq where up to 15 million Shia Muslims gather annually in Iraq for 15 days, Vaisakhi, which celebrates Sikh New Year, and the duodecennial Kumbh mela in India where 100 million Hindu pilgrims from all over India congregate over a 3-month period.^21^ Non-religious mass gatherings include the quadrennial Olympic Games and the Football World Cup. Mass gatherings increase the risk of the spread of respiratory tract infections.^22^^,^^23^ Pneumonia accounts for 20–39% of hospital admission during the Hajj, with an incidence of 4.8/100 000 and a case fatality rate of 34%.^24^ Over 60% of hospitalized cases of pneumonia were in adults aged >60 years and the CFR in those aged ≥50 years was 50%.^24^ In a study conducted in two hospitals in Makkah, during the 2004 Hajj, pneumonia was the cause of severe sepsis in 55% of hospital admissions and *Streptococcus pneumoniae* was identified in 18.75% of the cases.^25^ A review of studies of nasopharyngeal carriage of *Streptococcus pneumoniae* among pilgrims before and after Hajj found a significant increase significantly from 18% to 36%.^26^ There is clearly potential for the global spread of pneumococcal serotypes and antimicrobial resistance.^27^

We conducted a systematic search of publicly available data on the levels of pneumococcal infection (invasive and non-invasive), PCV vaccination implementation, duration of the PCV programme and PCV vaccine coverage in individual countries. In order to help support implementation of the JCVI recommendations, these data were then used to develop an algorithm using a pragmatic approach to identify UK travellers (particularly those aged over 65 years) who may be at increased risk of pneumococcal infection when travelling through, or seeking residence in, specific countries globally. By utilizing the latest country-specific data, this algorithm could be used to support national travel advisory bodies and other providers of travel vaccinations, with the option to be regularly updated.

## Methods

### Data sources

To ensure robust contemporary global data, we explored data sources from recognized established organizations routinely collecting information on pneumococcal disease, such as the WHO, European Centre for Disease Prevention and Control (ECDC) and Centres for Disease Control and Prevention (CDC). All data used for the development of the algorithm can be found in the Supplementary Data Tables provided. The WHO database was searched for individual country data on vaccine coverage and whether an infant PCV immunization programme was in place.^28^ Robust data sources for total cases of pneumococcal disease were identified through the CDC (USA) and ECDC (Europe).^29^^,^^30^ However, equivalent regional databases for the rest of the Americas, Eastern Mediterranean, South East Asia and Western Pacific were lacking, with no case or incidence data currently published independently by the WHO. The International Vaccine Access Centre (IVAC) Vaccine Information and Epidemiology Window (VIEW)-hub database provided global data for the level of pneumococcal infections by country to address this lack of data.^31^ The IVAC VIEW-hub database was searched for data on incidence of pneumococcal infection, duration of vaccine programme and the type of vaccine used in childhood vaccination programmes (PCV13, PCV10 or both).^31^ Data collection was initially performed in 2018. These numbers were updated in May 2020 to provide more recent figures for PCV coverage and programme duration, prior to manuscript submission. The WHO and IVAC VIEW-hub lack published data for some specific countries; in these situations an additional search of national public health websites and peer-reviewed literature was carried out.^31–36^ This included the *WHO vaccine-preventable diseases: monitoring system 2019 global summary*.^36^ Where data could not be found, countries were listed as ‘unknown’. All data used for the development of the algorithm can be found in the Supplementary Data Tables provided.

### Data collection

Data were collected in a fully filterable database and organized by the WHO region groupings: AFR—Africa; AMR—Americas; EMR—Eastern Mediterranean; EUR—Europe; SEAR—South East Asia; and WPR—Western Pacific.^37^ Data were collected for: ‘PCV coverage’, defined as the proportion of the infant population vaccinated with PCV; ‘PCV programme duration’, defined as the completed number of years since the PCV vaccination programme was introduced (completed years up to 1 May 2020); and ‘incidence’ or case number, defined as the total cases of pneumococcal infections (invasive and non-invasive) per 100 000/year.

Case numbers for pneumococcal disease, stratified by age, country and standardized globally are not available; therefore, a pragmatic and consistent approach was taken using the most recent case data from one main source (IVAC VIEW-hub platform; 2015 figures) and population estimates from a second source (*The World Factbook*, from the US Central Intelligence Agency).^31^^,^^38^ This ensures that data from different countries are comparable.

Cases per 100 000/year were calculated using the sum of all cases of pneumococcal infection per country in 2015, dividing by the estimated total population of that country and multiplying by 100 000.^31^^,^^38^ Total cases of pneumococcal infection were calculated across all age groups and included cases of severe pneumonia, pneumonia, pneumococcal meningitis, non-pneumonia non-meningitis (NPNM) and non-severe NPNM.^31^ As the data were captured in these categories, cases could not be split into invasive and non-IPD. If case numbers were not available for one or more of these categories, the country was given an ‘unknown’ status for incidence.

### Defining country risk

Countries were categorized using a traffic light system as RED (high-risk), AMBER (intermediate-risk) or GREEN (low-risk). For ‘level of pneumococcal disease’, RED = ≥250 cases/100 000, AMBER = 51–249 cases/100 000 and GREEN = ≤50 cases/100 000. For ‘PCV coverage’, RED = ≤50%, AMBER = 51–79% and GREEN = ≥80%. For ‘PCV programme duration’, RED = ≤2 years, AMBER = >2–<5 years and GREEN = ≥5 years.

Overall risk for each country was categorized using a traffic light system and calculated by combining level of pneumococcal disease, PCV coverage and PCV programme duration data using a scoring system, with each parameter given equal weighting. The algorithm scoring system was defined as follows:

- Cases of pneumococcal infection ≥250/100 000 (RED): 3 points
- Cases of pneumococcal infection 51–249/100 000 (AMBER): 2 points
- Cases of pneumococcal infection ≤50/100 000 (GREEN): 1 point
- PCV coverage ≤50% (RED) or no vaccine programme (BLACK): 3 points
- PCV coverage 51–79% (AMBER): 2 points
- PCV coverage ≥80% (GREEN): 1 point
- PCV programme duration ≤2 years (RED) or no vaccine programme (BLACK): 3 points
- PCV programme duration >2–<5 years (AMBER): 2 points
- PCV programme duration ≥5 years (GREEN): 1 point

When no pneumococcal conjugate vaccine programme was confirmed in a specific county, 3 points were scored for both PCV programme duration and coverage. To be classed as ‘RED’ for ‘overall risk’, countries must score ≥8 points or have ≥2 RED parameters. To be classed as ‘AMBER’ for ‘overall risk’, countries must score 4–7 points. To be classed as ‘GREEN’ for ‘overall risk’, countries must score 2–3 points. Where countries were missing data for two or more parameters, they were classified as ‘unknown’. Where countries were missing data for one parameter, they were classified as follows: ‘RED’ if both known parameters were RED; ‘AMBER’ if one or more of the known parameters were AMBER; ‘GREEN’ if the two known parameters were GREEN. The scoring system was based on a combination of published evidence and expert opinion.^39^^,^^40^ For example, the *Global Vaccine Action Plan*, published by the WHO in 2013, recommended a coverage target of at least 90% at a national level and at least 80% coverage across every district for all vaccines in national immunization programmes.^40^ The algorithm presented here has been developed with a pragmatic approach, choosing a coverage target of 80% and above to be considered low risk.

Following the May 2020 update for PCV coverage and programme duration, the overall risk scores were also updated. This demonstrates that the spreadsheet and risk categories can be updated relatively easily, making it a viable option for travel immunisers to keep records renewed.

### Developing the algorithm

**Figure 1:**
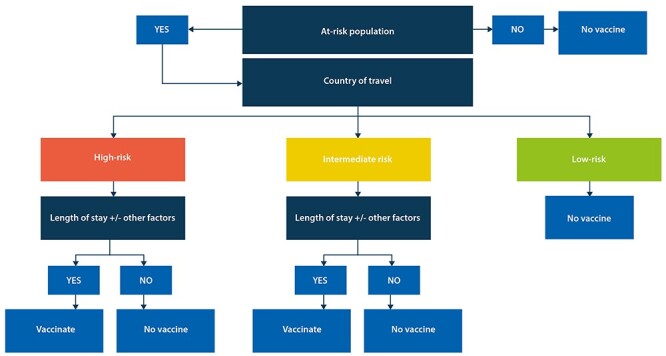
PCV travel algorithm. At-risk population: as defined in Chapter 25 of the *Green Book*. Country of travel: combines country-specific data to categorize potential risk of contracting pneumococcal infection per country. Length of stay ± other factors: to consider travel for more than 3–4 weeks, gathering in dense crowds, working with local communities, Hajj Pilgrimage, or travelling during ‘flu’ season

Using the data derived from the search, we developed an algorithm (Figure 1) using a stepwise approach to determine if PCV immunization should be recommended for travellers. Step 1 determines if the traveller falls into a population at increased risk of contracting pneumococcal infection. The population at increased risk includes clinical risk groups listed in the *Green Book* (Chapter 25: Immunisation Against Infectious Disease), an immunization guideline developed by Public Health England for healthcare professionals, for whom pneumococcal infection is likely to be more common and/or serious.^17^ This includes adults aged over 65 years and those with chronic disease including diabetes and heart disease.^17^ Step 2 determines whether the planned destination(s) include any countries where there is an increased risk of contracting IPD. Step 3 considers the specific context of an ‘at-risk individual’ travelling to a country where the risk of contracting a pneumococcal infection may be elevated.^41^ For example, individuals may require PCV13 vaccination if: travelling in a country for an extended period (>3–4 weeks); attending mass gatherings associated with dense crowds including international sporting events; working with local communities^16^; attending Hajj Pilgrimage^42^; or travelling during the ‘flu’ season (noting that this differs in northern and southern hemispheres). If a PCV10/13 vaccination was administered during the past 12 months then a second PCV13 vaccine would not be required.^6^^,^^43^ Responses following a second vaccination with PCV13 at 1 year have been demonstrated as non-inferior for a majority of serotypes compared with the initial PCV13 dose.^43^

## Results

### Burden of pneumococcal disease

**Figure 2:**
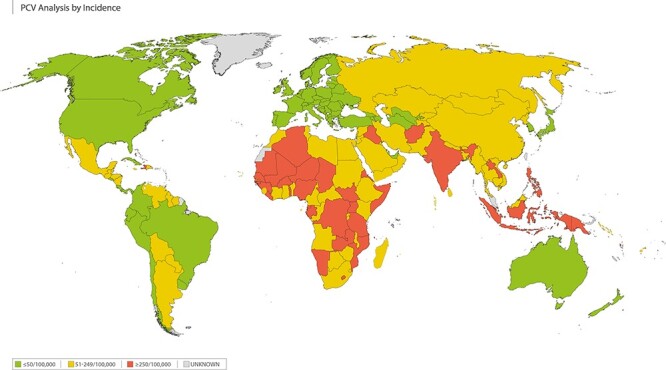
Level of pneumococcal disease (invasive and non-invasive)—cases per 100 000 population. igh level of disease (≥250 cases/100 000) shown in RED; intermediate level of disease (51–249 cases/100 000) shown in AMBER; low level of disease (≤50 cases/100 000) shown in GREEN; unknown level of disease shown in GREY

Case data were collated for 178 countries (Figure 2). A total of 35 countries had a ‘high’ case load of pneumococcal infection (incidence ≥250/100 000); 77 countries had ‘intermediate’ case load (incidence 51–249/100 000) and 66 countries were classified as ‘low’ case load (incidence ≤50/100 000). A total of 50 countries did not have all the individual case numbers required to calculate the total number of cases per 100 000 per year figure and, thus, were classed as ‘unknown’. (See Supplementary Data Tables for more details.)

### PCV coverage

**Figure 3:**
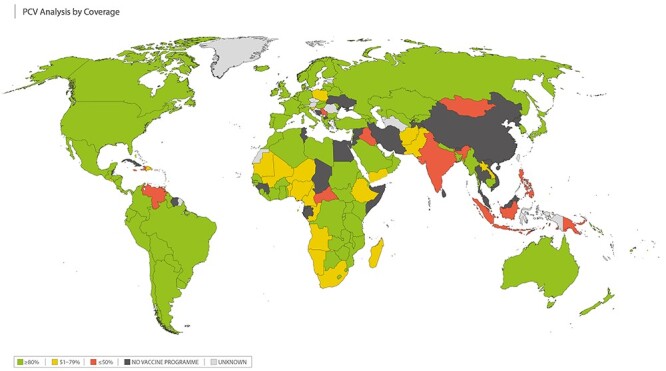
Pneumococcal conjugate vaccines (PCV) immunization coverage among 1 year olds (%). Low coverage (≤50%) shown in RED; intermediate coverage (51–79%) shown in AMBER; high coverage (≥80%) shown in GREEN; countries with no childhood vaccination programmes shown in BLACK; unknown coverage shown in GREY

We identified 144 countries as having an infant PCV immunization programme at the time of our analysis (Figure 3).^28^ Twelve of these countries were classified as having ‘low’ coverage (PCV coverage ≤50%); 23 countries were classified as ‘intermediate’ coverage (PCV coverage 51–79%); 109 countries were classified as ‘high’ coverage (PCV coverage ≥80%). We identified 42 countries who had no coverage data due to no vaccine programme at the time of our analysis and coverage data was unknown for 42 further countries. (See Supplementary Data Tables for more details.)

### PCV programme duration

**Figure 4:**
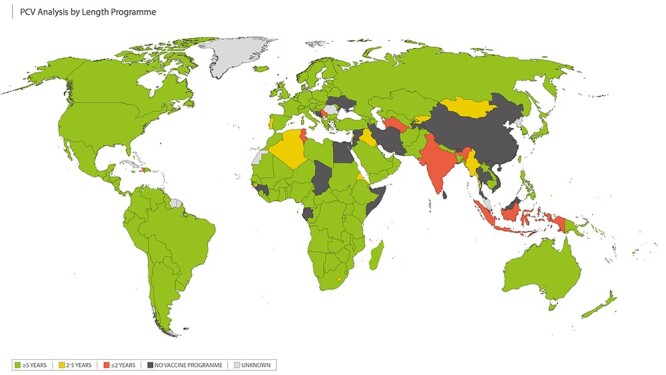
Time since PCV childhood vaccination programme was introduced (number of years). Short programme duration (≤2 years) shown in RED; intermediate programme duration (>2–<5 years) shown in AMBER; long programme duration (≥5 years) shown in GREEN; countries with no childhood vaccination programmes shown in BLACK; unknown programme duration shown in GREY

We collected PCV programme duration data for 154 countries, with most of the introduction dates taken from the IVAC VIEW-hub database (last updated 10 July 2018; accessed May 2020).^31^ PCV programme introduction was defined as countries that have introduced the vaccine nationally. Of these, 10 had a ‘short’ duration (≤2 years), 12 countries had an ‘intermediate’ programme duration (>2–<5 years) and 132 countries had a ‘long’ programme duration (≥5 years) (Figure 4). A total of 39 countries had no data to report at the time of our analysis and 35 countries had an unknown programme duration. (See Supplementary Data Tables for more details.)

### Overall risk

**Figure 5:**
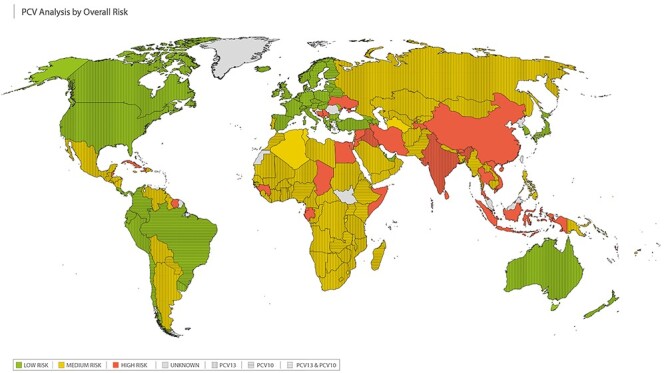
Data synthesis for the PCV travel vaccine algorithm; combining country-specific data to categorize potential risk of contracting pneumococcal infection. High overall risk (score ≥ 8 points or two RED parameters) shown in RED; intermediate overall risk (score 4–7 points) shown in AMBER; low overall risk shown in GREEN (score 2–3 points); unknown overall risk shown in GREY. Countries using PCV13 in childhood vaccination programmes visualized with vertical lines; countries using PCV10 in childhood vaccination programmes visualized with horizontal lines; and countries using both PCV13 and PCV10 in childhood vaccination programmes visualized with cross-hatched lines

We classified 45 countries as ‘high overall risk’ (RED; ≥8 points or have ≥2 RED parameters), 86 countries were classified as ‘intermediate overall risk’ (AMBER; 4–7 points) and 57 countries as ‘low overall risk’ (GREEN; 2–3 points) (Figure 5). A further 40 countries were listed as ‘unknown’ because we were missing data for ≥2 parameters.

It is worth noting that although Austria has had a PCV vaccine programme in place for many years, no coverage data are currently available. Austria was still considered low risk due to the duration and level of disease data. In addition, the vaccination programme start dates for Serbia and Tonga are later than the reported coverage figures and San Marino has coverage data for 2018 but no programme start date has been reported. A lower risk score may have been calculated if start dates were earlier than stated. These anomalies may be due to vaccinations occurring in some areas prior to the official start date and roll-out for the whole country or use in the private sector prior to introduction of the national immunization programme.

## Discussion

The introduction of PCVs has resulted in a substantial decline in pneumococcal infections around the world.^2^^,^^13^^,^^14^^,^^44^ However, there are still a large number of cases and deaths worldwide caused by vaccine-preventable serotypes of pneumococcus.^1^^,^^44^ Our study aimed to generate a list of countries where there is a high risk of contracting pneumococcal infection and to develop an algorithm to identify at-risk travellers who may require PCV13 vaccination prior to travelling to these countries. To our knowledge, this is the first attempt to pragmatically define the risk to travellers from the UK of contracting pneumococcal disease when travelling through, or seeking residence in, specific countries globally. Our algorithm employs a traffic light system, which is easy to follow and makes use of an established approach to categorize risk for other travel guidelines.^45^^,^^46^

By utilizing the latest country-specific data, this algorithm could be used by national travel advisory bodies and providers of travel vaccination. Data informing the algorithm should be updated regularly (e.g. every 3–5 years) to reflect the current environment in each country.

As healthcare systems and access to vaccines improve, we would expect more countries to move into the GREEN ‘low risk’ category over time. Childhood PCV vaccination programmes continue to be introduced worldwide, with coverage increasing across both high- and low-income countries.^40^ This action is partly driven by WHO recommendations and the *Global Vaccine Action Plan 2011–2020*.^40^^,^^47^

Data tables are included in the supplementary material and could be adapted to automatically update risk classification as data sources are updated.

Overall, we identified 36 countries with high level of pneumococcal disease (RED; incidence ≥250 cases/100 000), 13 countries with low PCV coverage (RED; coverage ≤50%), and 10 countries with short programme duration (RED; ≤2 years). Countries with low PCV coverage and short programme duration were evenly spread across WHO regions, however, a large concentration of high incidence countries (*n* = 25) were in the AFR region. Many countries in the AFR region have well-established childhood vaccination programmes (≥5 years) but despite this, all countries in this region had high (RED) or intermediate (AMBER) levels of pneumococcal infections. High overall risk countries were evenly spread across the different regions. In EMR, EUR, SEAR and WPR regions the reason for high overall risk was predominately due to countries having no PCV vaccination programmes in place. We listed 31 countries as ‘unknown’ in our database and these were predominately small nation countries including various Caribbean islands in AMR region and small islands in WPR region. As predicted, low overall risk countries (GREEN) were more likely to be the most developed or advanced countries (e.g. Australia, USA and many European countries). This could be because these countries have better access to vaccination programmes and, in general, have more advanced healthcare systems that lower the overall risk of infection.^48^^,^^49^

Infection levels and case numbers for pneumococcal disease should be interpreted with caution due to the limitations of the dataset. Epidemiological data often differs between countries.^50^ In many countries, high-quality mandatory monitoring systems are in place to record cases of pneumococcal disease; however, other countries will have less reliable surveillance systems. There may also be differences in medical practices, laboratory methods, case definitions or disease labelling.^51^ This may result in disparities in pneumococcal rates between countries.

A further limitation of this study is the possibility of underestimation of overall risk in certain countries. Data for one parameter were missing for several countries in our dataset and these countries were assigned a risk category based on the two known parameters. However, this approach, although pragmatic, could underestimate the risk evaluation for these countries.

In December 2019 the first cases of coronavirus disease 2019 (COVID-19) caused by severe acute respiratory syndrome coronavirus 2 (SARS-CoV-2) were reported in Wuhan, China with a global pandemic declared by WHO in March 2020.^52^ Varying social distancing measures have been implemented globally with a dramatic reduction in travel and incidence of IPD.^53^^,^^54^ A number of investigational vaccines against COVID-19 disease have now received emergency use or conditional approvals with the resulting mass vaccination programmes introduced in a number of countries. The impact of this global pandemic and mitigation measures, including social distancing and vaccination, on the risk of pneumococcal disease to travellers is unknown at this time. Early modelling suggests a potential increase in directly transmitted respiratory infections as travel and social distancing restrictions are lifted. In addition, the use of vaccine passports and a general willingness to travel post-pandemic may have influence on the risk of travel related disease. There are also recent interesting data suggesting PCV13 vaccination in older adults may have a positive impact on COVID-19 disease and diagnosis.^55^ The full impact of COVID-19 pandemic on travel related disease will become clearer as further data emerge. Despite these limitations, this is the first attempt to categorize the risk of contracting pneumococcal infection in each country, globally. These findings provide an evidence base to facilitate implementation of the JCVI recommendation and could be used by national travel advisory bodies and other providers of travel vaccines to identify adults at increased risk of pneumococcal infection whilst travelling. The lack of data on incidence of travel related pneumococcal disease is recognized and this algorithm may not be applicable to other countries where a routine PCV13 adult programme is recommended. Because this algorithm uses the latest country-specific data, it should be regularly updated, for example, every 3–5 years. In addition, this approach to categorizing risk could also be applied to other vaccine-preventable diseases.

## Supplementary Material

Supplementary_table_Coverage_estimates_200629_taab063

Supplementary_table_Incidence_200629_taab063

Supplementary_table_Vaccine_start_date_200630_taab063

## References

[1] Amin-Chowdhury Z, Iyanger N, Ramsay ME, Ladhani SN. Outbreaks of severe pneumococcal disease in closed settings in the conjugate vaccines era, 2010-2018: a systematic review to inform national guidance in the UK. J Infect 2019; 79:495–502. 31629865 10.1016/j.jinf.2019.10.009

[2] Martinez-Vega R, Jauneikaite E, Thoon KC et al. Risk factor profiles and clinical outcomes for children and adults with pneumococcal infections in Singapore: a need to expand vaccination policy? PLoS One 2019; 14:e0220951. 31618204 10.1371/journal.pone.0220951 PMC6795432

[3] Campling J, Jones D, Chalmers JD et al. The impact of certain underlying comorbidities on the risk of developing hospitalised pneumonia in England. Pneumonia (Nathan) 2019; 11:4. 31632897 10.1186/s41479-019-0063-z PMC6788086

[4] Merck Sharp & Dohme Corp . (Whitehouse Station, NJ, USA) Pneumovax 23. Prescribing Information. 2020. Available at: https://www.merck.com/product/usa/pi_circulars/p/pneumovax_23/pneumovax_pi.pdf (Accessed October 2020).

[5] GlaxoSmithKline . (Whitehouse Station, NJ, USA) Synflorix Summary of Product Characteristics. 2019. Available at: https://gskpro.com/content/dam/global/hcpportal/en_MT/PDF/Homepage/Products/synflorix/Synflorix%20SPC__(Oct_17).pdf (Accessed October 2020).

[6] Medicines.org.uk . Pfizer Limited. Prevanar 13 Summary of Product Characteristics. 2017. Available at: https://www.medicines.org.uk/emc/product/453/smpc (Accessed October 2020).

[7] Jackson LA, Gurtman A, Rice K et al. Immunogenicity and safety of a 13-valent pneumococcal conjugate vaccine in adults 70 years of age and older previously vaccinated with 23-valent pneumococcal polysaccharide vaccine. Vaccine 2013; 31:3585–93. 23688527 10.1016/j.vaccine.2013.05.010

[8] Moberley S, Holden J, Tatham DP, Andrews RM. Vaccines for preventing pneumococcal infection in adults. Cochrane Database Syst Rev 2013; 2013:CD000422. 10.1002/14651858.CD000422.pub3 PMC7045867 23440780

[9] Falkenhorst G, Remschmidt C, Harder T et al. Effectiveness of the 23-valent pneumococcal polysaccharide vaccine (PPV23) against pneumococcal disease in the elderly: systematic review and meta-analysis. PLoS One 2017; 12:e0169368. 28061505 10.1371/journal.pone.0169368 PMC5218810

[10] Papadatou I, Spoulou V. Pneumococcal vaccination in high-risk individuals: are we doing it right? Clin Vaccine Immunol 2016; 23:388–95. 27009210 10.1128/CVI.00721-15 PMC4860474

[11] Tomczyk S, Bennett NM, Stoecker C et al. Use of 13-valent pneumococcal conjugate vaccine and 23-valent pneumococcal polysaccharide vaccine among adults aged >/=65 years: recommendations of the advisory committee on immunization practices (ACIP). MMWR Morb Mortal Wkly Rep 2014; 63:822–5. 25233284 PMC5779453

[12] Papadatou I, Tzovara I, Licciardi PV. The role of serotype-specific immunological memory in pneumococcal vaccination: current knowledge and future prospects. Vaccines (Basel) 2019; 7:13. 10.3390/vaccines7010013 PMC6466264 30700048

[13] Hsu HE, Shutt KA, Moore MR et al. Effect of pneumococcal conjugate vaccine on pneumococcal meningitis. N Engl J Med 2009; 360:244–56. 19144940 10.1056/NEJMoa0800836 PMC4663990

[14] Kellner JD, Vanderkooi OG, MacDonald J et al. Changing epidemiology of invasive pneumococcal disease in Canada, 1998-2007: update from the Calgary-area Streptococcus pneumoniae research (CASPER) study. Clin Infect Dis 2009; 49:205–12. 19508165 10.1086/599827

[15] World Health Organisation . Immunization coverage. 2020. Available at: https://www.who.int/news-room/fact-sheets/detail/immunization-coverage (Accessed September 2020).

[16] Joint Committee on Vaccination and Immunisation . Meeting minutes. 2016. Available at: https://www.gov.uk/government/groups/joint-committee-on-vaccination-and-immunisation. (Accessed March 2020).

[17] Public Health England and Department of Health . Immunisation against Infectious Disease: Pneumococcal. Chapter 25. 2020. Available at: https://assets.publishing.service.gov.uk/government/uploads/system/uploads/attachment_data/file/857267/GB_Chapter_25_pneumococcal_January_2020.pdf (Accessed April 2021).

[18] Kingdom Saudi Arabia . |General Authority for Statistics (stats.gov.sa).

[19] Memish ZA, Assiri AM, Hussain R, Alomar I, Stephens G. Detection of respiratory viruses among pilgrims in Saudi Arabia during the time of a declared influenza A(H1N1) pandemic. J Travel Med 2012; 19:15–21. 22221807 10.1111/j.1708-8305.2011.00575.x PMC7537650

[20] Rashid H, Abdul Muttalif AR, Mohamed Dahlan ZB et al. The potential for pneumococcal vaccination in Hajj pilgrims: expert opinion. Travel Med Infect Dis 2013; 11:288–94. 23810307 10.1016/j.tmaid.2013.06.001

[21] Mass Gatherings & Global Health Network . (Accessed April 2021), at https://www.mghn.org/.

[22] Petersen E, Memish ZA, Zumla A, Maani AA. Transmission of respiratory tract infections at mass gathering events. Curr Opin Pulm Med 2020; 26:197–202. 32149751 10.1097/MCP.0000000000000675

[23] Alzeer AH . Respiratory tract infection during Hajj. Ann Thoracic Med 2009; 4:50–3. 10.4103/1817-1737.49412 PMC2700482 19561924

[24] Ghaznawi HIKM . Health hazards and risk factors in the 1406 (1986) Hajj season. Saudi Med J 1988; 9:274–82.

[25] Baharoon S, Al-Jahdali H, Al Hashmi J, Memish ZA, Ahmed QA. Severe sepsis and septic shock at the Hajj: Etiologies and outcomes. Travel Med Infect Dis 2009; 7:247–52. 19717109 10.1016/j.tmaid.2008.09.002

[26] Zafer N, Dulong C, Rahman A et al. Acute respiratory tract infection symptoms and the uptake of dual influenza and pneumococcal vaccines among Hajj pilgrims. Int Marit Health 2018; 69:278–84. 30589068 10.5603/IMH.2018.0044

[27] Memish ZA, Assiri A, Almasri M et al. Impact of the Hajj on pneumococcal transmission. Clin Microbiol Infect 2015; 21:e11–8. 25636939 10.1016/j.cmi.2014.07.005

[28] World Health Organisation . Global Health Observatory Data Repository: Pneumococcal conjugate (PCV3) immunization coverage estimates by country. 2020. Available at: http://apps.who.int/gho/data/node.main.PCV3n?lang=en (Accessed June 2020).

[29] Centers for Disease Control and Prevention . Pneumococcal Disease Surveillance and Reporting. 2017. Available at: https://www.cdc.gov/pneumococcal/surveillance.html (Accessed June 2020).

[30] European Centre for Disease Prevention and Control . Invasive pneumococcal disease - Annual epidemiological report for 2017. 2019. Available at: https://www.ecdc.europa.eu/en/publications-data/invasive-pneumococcal-disease-annual-epidemiological-report-2017 (Accessed July 2020).

[31] International Vaccine Access Center (IVAC) . VIEW-hub Data Visualization Platform. 2020. Available at: http://www.view-hub.org (Accessed June 2020).

[32] Taiwan Centers for Disease Control . Invasive pneumococcal disease. 2017. Available at: https://www.cdc.gov.tw/?aspxerrorpath=/english/info.aspx (Accessed June 2020).

[33] Hong Kong Centre for Health Protection . New pneumococcal vaccine for children immunisation programme programme. 2010. Available at: https://www.chp.gov.hk/en/features/21730.html (Accessed April 2020).

[34] Lee SY, Ieong KM. Pneumococcal infections pre and post vaccine era in Macao. J Paed Resp Crit Care 2016; 12:1–7.

[35] Tricarico S, McNeil HC, Cleary DW et al. Pneumococcal conjugate vaccine implementation in middle-income countries. Pneumonia (Nathan) 2017; 9:6. 28702308 10.1186/s41479-017-0030-5 PMC5471880

[36] World Health Organisation . WHO vaccine-preventable diseases: monitoring system. 2019 global summary. 2019. Available at: https://apps.who.int/immunization_monitoring/globalsummary (Accessed June 2020).

[37] World Health Organisation . Country groupings: Subregional country groupings for the global assessment of disease burden. 2020. Available at: http://www.who.int/quantifying_ehimpacts/global/ebdcountgroup/en/ (Accessed February 2020).

[38] Central Intelligence Agency , World Factbook. 2018. Available at: https://www.cia.gov/library/publications/the-world-factbook/ (Accessed July 2018).

[39] Aw B, Boraston S, Botten D et al. Travel medicine: what's involved? When to refer? Can Fam Physician 2014; 60:1091–103. 25500599 PMC4264804

[40] World Health Organisation . Global Vaccine Action Plan 2011-2020. 2013. Available at: https://www.who.int/immunization/global_vaccine_action_plan/GVAP_doc_2011_2020/en/ (Accessed June 2020).

[41] Centers for Disease Control and Prevention . Pneumococcal Disease (Streptococcus pneumoniae). Available at: https://wwwnc.cdc.gov/travel/diseases/pneumococcal-disease-streptococcus-pneumoniae (Accessed February 2020).

[42] Alqahtani AS, Tashani M, Ridda I et al. Burden of clinical infections due to S. pneumoniae during Hajj: a systematic review. Vaccine 2018; 36:4440–6. 29935859 10.1016/j.vaccine.2018.04.031

[43] Greenberg RN, Gurtman A, Frenck RW et al. Sequential administration of 13-valent pneumococcal conjugate vaccine and 23-valent pneumococcal polysaccharide vaccine in pneumococcal vaccine-naive adults 60-64 years of age. Vaccine 2014; 32:2364–74. 24606865 10.1016/j.vaccine.2014.02.002

[44] Wahl B, O'Brien KL, Greenbaum A et al. Burden of Streptococcus pneumoniae and Haemophilus influenzae type b disease in children in the era of conjugate vaccines: global, regional, and national estimates for 2000-15. Lancet Glob Health 2018; 6:e744–57. 29903376 10.1016/S2214-109X(18)30247-X PMC6005122

[45] Public Health England . Guidance: Rabies post-exposure treatment: management guidelines. 2013. Available at: https://www.gov.uk/government/publications/rabies-post-exposure-prophylaxis-management-guidelines (Accessed February 2020).

[46] European Centre for Disease prevention and Control . Maps in support of the Council Recommendation on a coordinated approach to travel measures in the EU. 2021. (Accessed April 2021, at https://www.ecdc.europa.eu/en/covid-19/situation-updates/weekly-maps-coordinated-restriction-free-movement.)

[47] World Health Organisation . Pneumococcal vaccines WHO position paper - 2012 - recommendations. Vaccine 2012; 30:4717–8. 22621828 10.1016/j.vaccine.2012.04.093

[48] World Health Organisation . Health care-associated infections Fact Sheet. 2019. Available at: https://www.who.int/gpsc/country_work/gpsc_ccisc_fact_sheet_en.pdf (Accessed December 2019).

[49] World Health Organisation . Preventing disease through healthy environments. 2018. Available at: https://www.who.int/quantifying_ehimpacts/publications/preventingdisease7.pdf (Accessed February 2020).

[50] Welte T, Kohnlein T. Global and local epidemiology of community-acquired pneumonia: the experience of the CAPNETZ Network. Semin Respir Crit Care Med 2009; 30:127–35. 19296412 10.1055/s-0029-1202941

[51] Sun X, Douiri A, Gulliford M. Pneumonia incidence trends in UK primary care from 2002 to 2017: population-based cohort study. Epidemiol Infect 2019; 147:e263. 31496464 10.1017/S0950268819001559 PMC6805760

[52] World Health Organization . Coronavirus disease 2019 (COVID-19) Situation Report - 51. 2020. Available at: https://www.who.int/docs/default-source/coronaviruse/situation-reports/20200311-sitrep-51-covid-19.pdf (Accessed April 2021).

[53] OECD Policy Responses to Coronavirus (COVID-19). Rebuilding tourism for the future: COVID-19 policy responses and recovery. 2020. (Accessed April 2021, at http://www.oecd.org/coronavirus/policy-responses/rebuilding-tourism-for-the-future-covid-19-policy-responses-and-recovery-bced9859/.)

[54] PubMLST . Invasive Respiratory Infections Surveillance (IRIS). 2021 Available at: https://pubmlst.org/projects/iris (Accessed April 2021).

[55] Lewnard JA, Bruxvoort KJ, Fischer H et al. Prevention of COVID-19 among older adults receiving pneumococcal conjugate vaccine suggests interactions between Streptococcus pneumoniae and SARS-CoV-2 in the respiratory tract. J Infect Dis 2021; jiab128. 10.1093/infdis/jiab128 33693636 PMC7989304

