# Supplementary material for: Identifying UK travellers at increased risk of developing pneumococcal infection: a novel algorithm
Source: J Travel Med. 2021 May 12;28(6):taab063. doi: 10.1093/jtm/taab063 (PMC8393689; doi:10.1093/jtm/taab063)
Supplement: Supplementary_table_Coverage_estimates_200629_taab063 [file supplementary_table_coverage_estimates_200629_taab063.pdf]

**Region** (WHO regional codes)	**Country** (2 letter country codes - country name)	**PCV coverage (%)**	**Data source**	**Date reported**
AFR	AO - Angola	67	WHO	2018
AFR	BF - Burkina Faso	91	WHO	2018
AFR	BI - Burundi	90	WHO	2018
AFR	BJ - Benin	73	WHO	2018
AFR	BW - Botswana	91	WHO	2018
AFR	CD - Democratic Republic of the Congo	81	WHO	2018
AFR	CF - Central African Republic	47	WHO	2018
AFR	CG - Congo	73	WHO	2018
AFR	CI - Côte d'Ivoire	81	WHO	2018
AFR	CM - Cameroon	79	WHO	2018
AFR	Comoros	NR	WHO	2018
AFR	CV - Cape Verde	NR	WHO	2018
AFR	DZ - Algeria	91	WHO	2018
AFR	ER - Eritrea	95	WHO	2018
AFR	ET - Ethiopia	67	WHO	2018
AFR	GA - Gabon	NR	WHO	2018
AFR	GH - Ghana	96	WHO	2018
AFR	GM - Gambia	93	WHO	2018
AFR	GN - Guinea	NR	WHO	2018
AFR	GQ - Equatorial Guinea	NR	WHO	2018
AFR	GW - Guinea-Bissau	88	WHO	2018
AFR	KE - Kenya	81	WHO	2018
AFR	LR - Liberia	84	WHO	2018
AFR	LS - Lesotho	93	WHO	2018
AFR	MG - Madagascar	75	WHO	2018
AFR	ML - Mali	68	WHO	2018
AFR	MR - Mauritania	77	WHO	2018

AFR	MU - Mauritius	96	WHO	2018
AFR	MW - Malawi	92	WHO	2018
AFR	MZ - Mozambique	80	WHO	2018
AFR	NA - Namibia	61	WHO	2018
AFR	NE - Niger	79	WHO	2018
AFR	NG - Nigeria	57	WHO	2018
AFR	RE - Reunion	NR	WHO	2018
AFR	RW - Rwanda	97	WHO	2018
AFR	SC - Seychelles	16	WHO	2018
AFR	SL - Sierra Leone	90	WHO	2018
AFR	SN - Senegal	81	WHO	2018
AFR	SO - Somalia	NR	WHO	2018
AFR	South Sudan	93	WHO	2018
AFR	ST - Sao Tome and Principe	95	WHO	2018
AFR	SZ - Swaziland	88	WHO	2018
AFR	TD - Chad	NR	WHO	2018
AFR	TG - Togo	88	WHO	2018
AFR	TZ - United Republic of Tanzania	98	WHO	2018
AFR	UG - Uganda	92	WHO	2018
AFR	Western Sahara	NR	WHO	2018
AFR	ZA - South Africa	73	WHO	2018
AFR	ZM - Zambia	90	WHO	2018
AFR	ZW - Zimbabwe	89	WHO	2018
AMR	AG - Antigua and Barbuda	NR	WHO	2018
AMR	AI - Anguilla	NR	WHO	2018
AMR	AR - Argentina	88	WHO	2018
AMR	AW - Aruba	NR	WHO	2018
AMR	BB - Barbados	89	WHO	2018
AMR	BM - Bermuda	NR	WHO	2018
AMR	BO - Bolivia	83	WHO	2018
AMR	BR - Brazil	84	WHO	2018
AMR	BS - Bahamas	90	WHO	2018
AMR	BZ - Belize	NR	WHO	2018

AMR	CA - Canada	81	WHO	2018
AMR	Caribbean Netherlands	NR	WHO	2018
AMR	CL - Chile	93	WHO	2018
AMR	CO - Columbia	94	WHO	2018
AMR	CR - Costa Rica	96	WHO	2018
AMR	CU - Cuba	NR	WHO	2018
AMR	CW - Curaçao	NR	WHO	2018
AMR	DM - Dominica	NR	WHO	2018
AMR	DO - Dominican Republic	70	WHO	2018
AMR	EC - Ecuador	85	WHO	2018
AMR	FK - Falkland Islands	NR	WHO	2018
AMR	GD - Grenada	NR	WHO	2018
AMR	GF - French Guiana	NR	WHO	2018
AMR	GP - Guadeloupe	NR	WHO	2018
AMR	Greenland	NR	WHO	2018
AMR	GT - Guatemala	85	WHO	2018
AMR	GY - Guyana	91	WHO	2018
AMR	HN - Honduras	90	WHO	2018
AMR	HT - Haiti	1	WHO	2018
AMR	JM - Jamaica	39	ViewHub/ Country-reported*	2017
AMR	KN - Saint Kitts and Nevis	NR	WHO	2018
AMR	KY - Cayman Islands	NR	WHO	2018
AMR	LC - Saint Lucia	NR	WHO	2018
AMR	Montserrat	NR	WHO	2018
AMR	MQ - Martinique	NR	WHO	2018
AMR	MX - Mexico	88	WHO	2018
AMR	NI - Nicaragua	98	WHO	2018
AMR	PA - Panama	92	WHO	2018
AMR	PE - Peru	82	WHO	2018
AMR	PR - Puerto Rico	NR	WHO	2018
AMR	PY - Paraguay	94	WHO	2018
AMR	SR - Suriname	NR	WHO	2018

AMR	SV - El Salvador	75	WHO	2018
AMR	SX - Sint Maarten	NR	WHO	2018
AMR	TC - Turks and Caicos	NR	WHO	2018
AMR	TT - Trinidad and Tobago	99	WHO	2018
AMR	United States Virgin Islands	NR	WHO	2018
AMR	United States of America	92	WHO	2018
AMR	UY - Uruguay	93	WHO	2018
AMR	VC - Saint Vincent and the Grenadines	NR	WHO	2018
AMR	VE - Venezuela	7	WHO	2016
AMR	VG - British Virgin Islands	NR	WHO	2018
EUR	AD - Andorra	94	WHO	2018
EUR	AL - Albania	98	WHO	2018
EUR	AM - Armenia	92	WHO	2018
EUR	AT - Austria	NR	WHO	2018
EUR	AZ - Azerbaijan	95	WHO	2018
EUR	BA - Bosnia and Herzegovina	NR	WHO	2018
EUR	BE - Belgium	94	WHO	2018
EUR	BG - Bulgaria	88	WHO	2018
EUR	BY - Belarus	98	ViewHub/ Country-reported*	2017
EUR	CH - Switzerland	85	WHO	2018
EUR	CY - Cyprus	81	WHO	2018
EUR	CZ - Czech Republic	NR	WHO	2018
EUR	DE - Germany	84	WHO	2018
EUR	DK - Denmark	96	WHO	2018
EUR	EE - Estonia	NR	WHO	2018
EUR	ES - Spain	93	WHO	2018
EUR	FI - Finland	88	WHO	2018
EUR	FO - Faroe Islands	NR	WHO	2018
EUR	FR - France	92	WHO	2018
EUR	GE - Georgia	81	WHO	2018
EUR	GI - Gibraltar	NR	WHO	2018

EUR	GR - Greece	96	WHO	2018
EUR	HR - Croatia	NR	WHO	2018
EUR	HU - Hungary	99	WHO	2018
EUR	IE - Ireland	90	WHO	2018
EUR	IL - Israel	94	WHO	2018
EUR	IS - Iceland	90	WHO	2018
EUR	IT - Italy	92	WHO	2018
EUR	KG - Kyrgyzstan	92	WHO	2018
EUR	KZ - Kazakhstan	95	WHO	2018
EUR	LI - Liechtenstein	NR	WHO	2018
EUR	LT - Lithuania	82	WHO	2018
EUR	LU - Luxembourg	96	WHO	2018
EUR	LV - Latvia	82	WHO	2018
EUR	MC - Monaco	NR	WHO	2018
EUR	MD - Republic of Moldova	94	WHO	2018
EUR	ME - Montenegro	NR	WHO	2018
EUR	MK - Macedonia	NR	WHO	2018
EUR	MT - Malta	NR	WHO	2018
EUR	NL - Netherlands	93	WHO	2018
EUR	NO - Norway	94	WHO	2018
EUR	PL - Poland	60	WHO	2018
EUR	PT - Portugal	98	WHO	2018
EUR	RO - Romania	NR	WHO	2018
EUR	RS - Serbia	48	WHO	2018
EUR	RU - Russian Federation	82	WHO	2018
EUR	SE - Sweden	97	WHO	2018
EUR	SI - Slovenia	60	WHO	2018
EUR	SK - Slovakia	96	WHO	2018
EUR	SM - San Marino	58	WHO	2018
EUR	TR - Turkey	97	WHO	2018
EUR	UA - Ukraine	NR	WHO	2018
EUR	UK - United Kingdom	92	WHO	2018
EUR	UZ - Uzbekistan	96	WHO	2018

EUR	XK - Kosovo	NR	WHO	2018
EMR	AE - United Arab Emirates	99	WHO	2018
EMR	AF - Afghanistan	65	WHO	2018
EMR	BH - Bahrain	98	WHO	2018
EMR	DJ - Djibouti	84	WHO	2018
EMR	EG - Egypt	NR	WHO	2018
EMR	IQ - Iraq	32	WHO	2018
EMR	IR - Iran (Islamic Republic of)	NR	WHO	2018
EMR	JO - Jordan	NR	WHO	2018
EMR	KW - Kuwait	99	WHO	2018
EMR	LB - Lebanon	82	WHO	2018
EMR	LY - Libya	96	WHO	2018
EMR	MA - Morocco	99	WHO	2018
EMR	OM - Oman	99	WHO	2018
EMR	PK - Pakistan	79	WHO	2018
EMR	QA - Qatar	98	WHO	2018
EMR	SA - Saudi Arabia	98	WHO	2018
EMR	SD - Sudan	93	WHO	2018
EMR	SY - Syria	NR	WHO	2018
EMR	TJ - Tajikistan	NR	WHO	2018
EMR	TM - Turkmenistan	NR	WHO	2018
EMR	TN - Tunisia	NR	WHO	2018
EMR	YE - Yemen	64	WHO	2018
SEAR	BD - Bangladesh	97	WHO	2018
SEAR	BT - Bhutan	NR	WHO	2018
SEAR	ID - Indonesia	8	WHO	2018
SEAR	IN - India	6	WHO	2018
SEAR	KP - North Korea	NR	WHO	2018
SEAR	KR - South Korea	97	WHO	2018
SEAR	LK - Sri Lanka	NR	WHO	2018
SEAR	MM - Myanmar	91	WHO	2018
SEAR	MV - Maldives	NR	WHO	2018
SEAR	MY - Malaysia	NR	WHO	2018

SEAR	NP - Nepal	82	WHO	2018
SEAR	TH - Thailand	NR	WHO	2018
SEAR	TL - Timor Leste	NR	WHO	2018
WPR	AS - American Samoa	NR	WHO	2018
WPR	AU - Australia	95	WHO	2018
WPR	BN - Brunei Darussalam	NR	WHO	2018
WPR	CK - Cook Islands	NR	WHO	2018
WPR	CN - China	NR	WHO	2018
WPR	FJ - Fiji	99	WHO	2018
WPR	FM - Micronesia	67	WHO	2018
WPR	GU - Guam	NR	WHO	2018
WPR	HK - Hong Kong	NR	WHO	2018
WPR	JP - Japan	98	WHO	2018
WPR	KH - Cambodia	84	WHO	2018
WPR	KI - Kiribati	94	WHO	2018
WPR	LA - Lao People's Democratic Republic	56	WHO	2018
WPR	MH - Marshall Islands	67	WHO	2018
WPR	MN - Mongolia	26	WHO	2018
WPR	MO - Macao	NR	WHO	2018
WPR	MP - Northern Mariana Islands	NR	WHO	2018
WPR	NC - New Caledonia	NR	WHO	2018
WPR	NR - Nauru	NR	WHO	2018
WPR	NU - Niue	99	WHO	2018
WPR	NZ - New Zealand	96	WHO	2018
WPR	PF - French Polynesia	NR	WHO	2018
WPR	PG - Papua New Guinea	43	WHO	2018
WPR	PH - Philippines	43	WHO	2018
WPR	PN - Pitcairn Islands	NR	WHO	2018
WPR	PW - Palau	89	WHO	2018
WPR	SB - Solomon Islands	84	WHO	2018
WPR	SG - Singapore	82	WHO	2018
WPR	TK - Tokelau	NR	WHO	2018

WPR	TO - Tonga	99	ViewHub/ Country-reported*	2017
WPR	TV - Tuvalu	NR	WHO	2018
WPR	TW - Taiwan	NR	WHO	2018
WPR	VN - Vietnam	NR	WHO	2018
WPR	VU - Vanuatu	NR	WHO	2018
WPR	WF - Wallis and Futuna	NR	WHO	2018
WPR	WS - Samoa	NR	WHO	2018

NR - not reported
WHO - Taken from the WHO Global Health Observatory Database (last updated 26 August 2019)
*ViewHub/Country-reported - coverage estimates reported by national authorities through the WHO and UNICEF Join Reporting Forum (updated 18 July 2018)
