# Supplementary material for: Identifying UK travellers at increased risk of developing pneumococcal infection: a novel algorithm
Source: J Travel Med. 2021 May 12;28(6):taab063. doi: 10.1093/jtm/taab063 (PMC8393689; doi:10.1093/jtm/taab063)
Supplement: Supplementary_table_Incidence_200629_taab063 [file supplementary_table_incidence_200629_taab063.pdf]

Region (WHO regional codes)	Country (2 letter country codes - country name)	Country population	Data source	Date reported	Number of Non-severe NPNM cases	Number of NPNM cases	Number of Meningitis cases	Number of Severe pneumonia cases	Number of Pneumonia Cases	Total incidence of PD	Data source (ViewHub last updated July 2015)	Date reported	Incidence per 100,000
AFR	AO - Angola	29310273	CIA	Jul-17	3999	531	1324	15971	40976	62801	ViewHub	2015	214.26
AFR	BF - Burkina Faso	20107509	CIA	Jul-17	2198	292	728	14894	37869	55981	ViewHub	2015	278.41
AFR	BI - Burundi	11466756	CIA	Jul-17	1237	164	410	4993	12721	19525	ViewHub	2015	170.27
AFR	BJ - Benin	11038805	CIA	Jul-17	588	78	195	6559	16758	24178	ViewHub	2015	219.03
AFR	BW - Botswana	2214858	CIA	Jul-17	21	8	7	1047	2677	3760	ViewHub	2015	169.76
AFR	CD - Democratic Republic of the Congo	83301151	CIA	Jul-17	11106	1475	3678	73474	186642	276375	ViewHub	2015	331.78
AFR	CF - Central African Republic	5625118	CIA	Jul-17	758	101	251	3357	8561	13028	ViewHub	2015	231.60
AFR	CG - Congo	4954674	CIA	Jul-17	136	51	45	2788	7100	10120	ViewHub	2015	204.25
AFR	CI - Côte d'Ivoire	24184810	CIA	Jul-17	1569	208	520	10768	27641	40706	ViewHub	2015	168.31
AFR	CM - Cameroon	24994885	CIA	Jul-17	986	131	327	10042	25684	37170	ViewHub	2015	148.71
AFR	Comoros	808080	CIA	Jul-17	61	23	20	1016	2586	3706	ViewHub	2015	458.62
AFR	CV - Cape Verde	560899	CIA	Jul-17	53	20	18	335	856	1282	ViewHub	2015	228.56
AFR	DZ - Algeria	40969443	CIA	Jul-17	759	286	251	29391	74877	105564	ViewHub	2015	257.67
AFR	ER - Eritrea	5918919	CIA	Jul-17	488	183	161	5541	14161	20534	ViewHub	2015	346.92
AFR	ET - Ethiopia	105350020	CIA	Jul-17	3292	1238	1090	51106	130620	187346	ViewHub	2015	177.83
AFR	GA - Gabon	1722255	CIA	Jul-17	69	26	23	1672	4268	6058	ViewHub	2015	351.75
AFR	GH - Ghana	27499924	CIA	Jul-17	931	350	308	11106	28407	41102	ViewHub	2015	149.46
AFR	GM - Gambia	2051363	CIA	Jul-17	117	44	39	791	2020	3011	ViewHub	2015	146.78
AFR	GN - Guinea	12413867	CIA	Jul-17	1854	246	614	14083	36091	52888	ViewHub	2015	426.04
AFR	GQ - Equatorial Guinea	778358	CIA	Jul-17	53	7	17	847	2161	3085	ViewHub	2015	396.35
AFR	GW - Guinea-Bissau	1792338	CIA	Jul-17	245	33	81	1971	5033	7363	ViewHub	2015	410.80
AFR	KE - Kenya	47615739	CIA	Jul-17	1515	570	502	21076	53799	77462	ViewHub	2015	162.68
AFR	LR - Liberia	4689021	CIA	Jul-17	503	189	167	3154	8155	12168	ViewHub	2015	259.50
AFR	LS - Lesotho	1958042	CIA	Jul-17	253	34	84	1906	4865	7142	ViewHub	2015	364.75
AFR	MG - Madagascar	25054161	CIA	Jul-17	1359	511	450	13192	33711	49223	ViewHub	2015	196.47
AFR	ML - Mali	17885245	CIA	Jul-17	2226	296	737	16003	40950	60212	ViewHub	2015	336.66
AFR	MR - Mauritania	3758571	CIA	Jul-17	281	37	93	2611	6654	9676	ViewHub	2015	257.44
AFR	MU - Mauritius	1356388	CIA	Jul-17	18	7	6	452	1152	1635	ViewHub	2015	120.54
AFR	MW - Malawi	19196246	CIA	Jul-17	1661	625	550	8724	22218	33778	ViewHub	2015	175.96
AFR	MZ - Mozambique	26573706	CIA	Jul-17	2319	308	768	24789	62930	91114	ViewHub	2015	342.87
AFR	NA - Namibia	2484780	CIA	Jul-17	101	38	33	1837	4676	6685	ViewHub	2015	269.04
AFR	NE - Niger	19245344	CIA	Jul-17	4873	647	1614	27477	70460	105071	ViewHub	2015	545.96
AFR	NG - Nigeria	190632261	CIA	Jul-17	21497	2855	7119	254709	664724	950904	ViewHub	2015	498.82
AFR	RE - Reunion	Unknown											
AFR	RW - Rwanda	11901484	CIA	Jul-17	242	91	80	2307	5908	8628	ViewHub	2015	72.50
AFR	SC - Seychelles	93920	CIA	Jul-17	3	1	1	54	137	196	ViewHub	2015	208.69
AFR	SL - Sierra Leone	6163195	CIA	Jul-17	624	83	207	2590	6617	10121	ViewHub	2015	164.22
AFR	SN - Senegal	14668522	CIA	Jul-17	682	256	226	11614	29710	42488	ViewHub	2015	289.65
AFR	SO - Somalia	11031386	CIA	Jul-17	3393	451	1124	25887	66153	97008	ViewHub	2015	879.38
AFR	South Sudan	13026129	CIA	Jul-17	1884	250	624	10383	26689	39830	ViewHub	2015	305.77
AFR	ST - Sao Tome and Principe	201025	CIA	Jul-17	10	4	3	89	227	333	ViewHub	2015	165.65
AFR	SZ - Swaziland	1467152	CIA	Jul-17	62	23	21	564	1448	2118	ViewHub	2015	144.36
AFR	TD - Chad	12075985	CIA	Jul-17	4361	579	1444	17656	45472	69512	ViewHub	2015	575.62
AFR	TG - Togo	7965055	CIA	Jul-17	625	83	207	5742	14667	21324	ViewHub	2015	267.72
AFR	TZ - United Republic of Tanzania	53950935	CIA	Jul-17	3900	1467	1292	36429	92663	135751	ViewHub	2015	251.62
AFR	UG - Uganda	39570125	CIA	Jul-17	5854	2202	1939	36220	92487	138702	ViewHub	2015	350.52

Est - estimated

AFR	Western Sahara	603253	CIA	Jul-17	N/A	N/A	N/A	N/A	N/A	N/A	N/A	N/A	Unknown
AFR	ZA - South Africa	54841552	CIA	Jul-17	781	294	259	15994	40805	58133	ViewHub	2015	106.00
AFR	ZM - Zambia	15972000	CIA	Jul-17	1452	546	481	15597	39873	57949	ViewHub	2015	362.82
AFR	ZW - Zimbabwe	13805084	CIA	Jul-17	1193	449	395	9092	23202	34331	ViewHub	2015	248.68
AMR	AG - Antigua and Barbuda	94731	CIA	Jul-17	NR	NR	NR	26	66	92	ViewHub	2015	Unknown
AMR	AI - Anguilla	17087	CIA	Jul-17	N/A	N/A	N/A	N/A	N/A	N/A	N/A	N/A	Unknown
AMR	AR - Argentina	44293293	CIA	Jul-17	647	243	214	6543	16778	24425	ViewHub	2015	55.14
AMR	AW - Aruba	115120	CIA	Jul-17	N/A	N/A	N/A	N/A	N/A	N/A	N/A	N/A	Unknown
AMR	BB - Barbados	292336	CIA	Jul-17	NR	NR	NR	19	49	68	ViewHub	2015	Unknown
AMR	BM - Bermuda	70864	CIA	Jul-17	N/A	N/A	N/A	N/A	N/A	N/A	N/A	N/A	Unknown
AMR	BO - Bolivia	11138234	CIA	Jul-17	129	49	43	2724	6984	9929	ViewHub	2015	89.14
AMR	BR - Brazil	207353391	CIA	Jul-17	546	205	181	22494	57716	81142	ViewHub	2015	39.13
AMR	BS - Bahamas	329988	CIA	Jul-17	2	1	1	32	82	118	ViewHub	2015	35.76
AMR	BZ - Belize	360346	CIA	Jul-17	14	5	5	186	476	686	ViewHub	2015	190.37
AMR	CA - Canada	35623680	CIA	Jul-17	63	24	21	234	360	702	ViewHub	2015	1.97
AMR	Caribbean Netherlands	Unknown											
AMR	CL - Chile	17789267	CIA	Jul-17	111	42	37	1657	4256	6103	ViewHub	2015	34.31
AMR	CO - Columbia	47698524	CIA	Jul-17	167	63	55	4449	11394	16128	ViewHub	2015	33.81
AMR	CR - Costa Rica	4930258	CIA	Jul-17	9	3	3	365	938	1318	ViewHub	2015	26.73
AMR	CU - Cuba	11147407	CIA	Jul-17	153	58	51	862	1329	2453	ViewHub	2015	22.01
AMR	CW - Curaçao	149648	CIA	Jul-17	N/A	N/A	N/A	N/A	N/A	N/A	N/A	N/A	Unknown
AMR	DM - Dominica	73897	CIA	Jul-17	NR	NR	NR	22	56	78	ViewHub	2015	Unknown
AMR	DO - Dominican Republic	10734247	CIA	Jul-17	101	38	33	4069	10433	14674	ViewHub	2015	136.70
AMR	EC - Ecuador	16290913	CIA	Jul-17	641	241	212	1881	4822	7797	ViewHub	2015	47.86
AMR	FK - Falkland Islands	2931	CIA	2014	N/A	N/A	N/A	N/A	N/A	N/A	N/A	N/A	Unknown
AMR	GD - Grenada	111724	CIA	Jul-17	NR	NR	NR	35	91	126	ViewHub	2015	Unknown
AMR	GF - French Guiana	Unknown											
AMR	GP - Guadeloupe	Unknown											
AMR	Greenland	57713	CIA	Jul-17	N/A	N/A	N/A	N/A	N/A	N/A	N/A	N/A	Unknown
AMR	GT - Guatemala	15460732	CIA	Jul-17	291	109	96	6465	16539	23500	ViewHub	2015	152.00
AMR	GY - Guyana	737718	CIA	Jul-17	13	5	4	142	363	527	ViewHub	2015	71.44
AMR	HN - Honduras	9038741	CIA	Jul-17	38	14	13	1349	3451	4865	ViewHub	2015	53.82
AMR	HT - Haiti	10646714	CIA	Jul-17	738	278	244	7899	20282	29441	ViewHub	2015	276.53
AMR	JM - Jamaica	2990561	CIA	Jul-17	24	9	8	725	1861	2627	ViewHub	2015	87.84
AMR	KN - Saint Kitts and Nevis	52715	CIA	Jul-17	5	2	2	16	42	67	ViewHub	2015	127.10
AMR	KY - Cayman Islands	58441	CIA	Jul-17	N/A	N/A	N/A	N/A	N/A	N/A	N/A	N/A	Unknown
AMR	LC - Saint Lucia	164994	CIA	Jul-17	6	2	2	49	126	185	ViewHub	2015	112.13
AMR	Montserrat	5292	CIA	Jul-17	N/A	N/A	N/A	N/A	N/A	N/A	N/A	N/A	Unknown
AMR	MQ - Martinique	Unknown											
AMR	MX - Mexico	124574795	CIA	Jul-17	1828	688	606	16837	43214	63173	ViewHub	2015	50.71
AMR	NI - Nicaragua	6025951	CIA	Jul-17	42	16	14	898	2298	3268	ViewHub	2015	54.23
AMR	PA - Panama	3753142	CIA	Jul-17	27	10	9	432	1107	1585	ViewHub	2015	42.23
AMR	PE - Peru	31036656	CIA	Jul-17	158	60	52	3247	8362	11879	ViewHub	2015	38.27
AMR	PR - Puerto Rico	3351827	CIA	Jul-17	N/A	N/A	N/A	N/A	N/A	N/A	N/A	N/A	Unknown
AMR	PY - Paraguay	6943739	CIA	Jul-17	281	106	93	1506	3857	5843	ViewHub	2015	84.15
AMR	SR - Suriname	591919	CIA	Jul-17	18	7	6	171	440	642	ViewHub	2015	108.46
AMR	SV - El Salvador	6172011	CIA	Jul-17	33	12	11	672	1723	2451	ViewHub	2015	39.71
AMR	SX - Sint Maarten	42083	CIA	Jul-17	N/A	N/A	N/A	N/A	N/A	N/A	N/A	N/A	Unknown
AMR	TC - Turks and Caicos	52570	CIA	Jul-17	N/A	N/A	N/A	N/A	N/A	N/A	N/A	N/A	Unknown
AMR	TT - Trinidad and Tobago	1218208	CIA	Jul-17	6	2	2	167	429	606	ViewHub	2015	49.75
AMR	United States Virgin Islands	107268	CIA	Jul-17	N/A	N/A	N/A	N/A	N/A	N/A	N/A	N/A	Unknown

AMR	US - United States of America	326625791	CIA	Jul-17	353	133	117	2369	3654	6626	ViewHub	2015	2.03
AMR	UY - Uruguay	3360148	CIA	Jul-17	31	12	10	231	594	878	ViewHub	2015	26.13
AMR	VC - Saint Vincent and the Grenadines	102089	CIA	Jul-17	NR	NR	NR	30	78	108	ViewHub	2015	Unknown
AMR	VE - Venezuela	31304016	CIA	Jul-17	414	156	137	9890	25362	35959	ViewHub	2015	114.87
AMR	VG - British Virgin Islands	35015	CIA	Jul-17	N/A	N/A	N/A	N/A	N/A	N/A	N/A	N/A	Unknown
EMR	AE - United Arab Emirates	6072475	CIA	Jul-17	22	8	7	673	1729	2439	ViewHub	2015	40.16
EMR	AF - Afghanistan	34124811	CIA	Jul-17	3856	512	1277	50138	127148	182931	ViewHub	2015	536.06
EMR	BH - Bahrain	1410942	CIA	Jul-17	12	4	4	136	350	506	ViewHub	2015	35.86
EMR	DJ - Djibouti	865267	CIA	Jul-17	81	31	27	333	849	1321	ViewHub	2015	152.67
EMR	EG - Egypt	97041072	CIA	Jul-17	1373	516	455	39826	102056	144226	ViewHub	2015	148.62
EMR	IQ - Iraq	39192111	CIA	Jul-17	1047	394	347	31294	79601	112683	ViewHub	2015	287.51
EMR	IR - Iran (Islamic Republic of)	82021564	CIA	Jul-17	756	284	250	28173	72319	101782	ViewHub	2015	124.09
EMR	JO - Jordan	10248069	CIA	Jul-17	124	47	41	4022	10345	14579	ViewHub	2015	142.26
EMR	KW - Kuwait	2875422	CIA	Jul-17	54	20	18	436	1120	1648	ViewHub	2015	57.31
EMR	LB - Lebanon	6229794	CIA	Jul-17	21	8	7	1895	4868	6799	ViewHub	2015	109.14
EMR	LY - Libya	6653210	CIA	Jul-17	155	58	51	2218	5697	8179	ViewHub	2015	122.93
EMR	MA - Morocco	33986655	CIA	Jul-17	264	99	87	6743	17180	24373	ViewHub	2015	71.71
z	OM - Oman	3424386	CIA	Jul-17	45	17	15	496	1275	1848	ViewHub	2015	53.97
EMR	PK - Pakistan	204924861	CIA	Jul-17	9355	1243	3098	132256	339428	485380	ViewHub	2015	236.86
EMR	QA - Qatar	2314307	CIA	Jul-17	9	3	3	172	442	629	ViewHub	2015	27.18
EMR	SA - Saudi Arabia	28571770	CIA	Jul-17	418	157	138	4085	10474	15272	ViewHub	2015	53.45
EMR	SD - Sudan	37345935	CIA	Jul-17	2141	805	709	18532	47186	69373	ViewHub	2015	185.76
EMR	SY - Syria	18028549	CIA	Jul-17	233	88	77	9015	23133	32546	ViewHub	2015	180.52
EMR	TJ - Tajikistan	8468555	CIA	Jul-17	678	255	224	1724	2659	5540	ViewHub	2015	65.42
EMR	TM - Turkmenistan	5351277	CIA	Jul-17	195	73	65	774	1194	2301	ViewHub	2015	43.00
EMR	TN - Tunisia	11403800	CIA	Jul-17	50	19	17	4035	10365	14486	ViewHub	2015	127.03
EMR	YE - Yemen	28036829	CIA	Jul-17	335	126	111	9197	23439	33208	ViewHub	2015	118.44
EUR	AD - Andorra	85702	CIA	Jul-17	NR	NR	NR	1	1	2	ViewHub	2015	Unknown
EUR	AL - Albania	3047987	CIA	Jul-17	7	3	2	84	129	225	ViewHub	2015	7.38
EUR	AM - Armenia	3045191	CIA	Jul-17	39	15	13	259	399	725	ViewHub	2015	23.81
EUR	AT - Austria	8754413	CIA	Jul-17	46	17	15	296	457	831	ViewHub	2015	9.49
EUR	AZ - Azerbaijan	9961396	CIA	Jul-17	71	27	23	867	1337	2325	ViewHub	2015	23.34
EUR	BA - Bosnia and Herzegovina	3856181	CIA	Jul-17	3	1	1	253	390	648	ViewHub	2015	16.80
EUR	BE - Belgium	11491346	CIA	Jul-17	34	13	11	198	306	562	ViewHub	2015	4.89
EUR	BG - Bulgaria	7101510	CIA	Jul-17	27	10	9	142	220	408	ViewHub	2015	5.75
EUR	BY - Belarus	9549747	CIA	Jul-17	29	11	10	1719	2652	4421	ViewHub	2015	46.29
EUR	CH - Switzerland	8236303	CIA	Jul-17	2	1	1	81	125	210	ViewHub	2015	2.55
EUR	CY - Cyprus	1221549	CIA	Jul-17	3	1	1	71	110	186	ViewHub	2015	15.23
EUR	CZ - Czech Republic	10674723	CIA	Jul-17	42	16	14	394	608	1074	ViewHub	2015	10.06
EUR	DE - Germany	80594017	CIA	Jul-17	39	15	13	407	628	1102	ViewHub	2015	1.37
EUR	DK - Denmark	5605948	CIA	Jul-17	3	1	1	47	72	124	ViewHub	2015	2.21
EUR	EE - Estonia	1251581	CIA	Jul-17	7	3	2	211	326	549	ViewHub	2015	43.86
EUR	ES - Spain	48958159	CIA	Jul-17	173	65	57	1572	2425	4292	ViewHub	2015	8.77
EUR	FI - Finland	5518371	CIA	Jul-17	4	2	1	108	166	281	ViewHub	2015	5.09
EUR	FO - Faroe Islands	50730	CIA	Jul-17	N/A	N/A	N/A	N/A	N/A	N/A	N/A	N/A	Unknown
EUR	FR - France	67106161	CIA	Jul-17	101	38	33	472	728	1372	ViewHub	2015	2.04
EUR	GE - Georgia	4926330	CIA	Jul-17	18	7	6	382	590	1003	ViewHub	2015	20.36
EUR	GI - Gibraltar	29396	CIA	Jul-17	N/A	N/A	N/A	N/A	N/A	N/A	N/A	N/A	Unknown
EUR	GR - Greece	10768477	CIA	Jul-17	3	1	1	150	231	386	ViewHub	2015	3.58
EUR	HR - Croatia	4292095	CIA	Jul-17	36	13	12	152	234	447	ViewHub	2015	10.41

EUR	HU - Hungary	9850845	CIA	Jul-17	17	6	6	387	597	1013	ViewHub	2015	10.28
EUR	IE - Ireland	5011102	CIA	Jul-17	10	4	3	61	94	172	ViewHub	2015	3.43
EUR	IL - Israel	8299706	CIA	Jul-17	55	21	18	221	341	656	ViewHub	2015	7.90
EUR	IS - Iceland	339747	CIA	Jul-17	NR	NR	NR	8	13	21	ViewHub	2015	Unknown
EUR	IT - Italy	62137802	CIA	Jul-17	20	8	7	371	573	979	ViewHub	2015	1.58
EUR	KG - Kyrgyzstan	5789122	CIA	Jul-17	165	62	55	1144	1765	3191	ViewHub	2015	55.12
EUR	KZ - Kazakhstan	18556698	CIA	Jul-17	172	65	57	3653	5635	9582	ViewHub	2015	51.64
EUR	LI - Liechtenstein	38244	CIA	Jul-17	N/A	N/A	N/A	N/A	N/A	N/A	N/A	N/A	Unknown
EUR	LT - Lithuania	2823859	CIA	Jul-17	51	19	17	445	687	1219	ViewHub	2015	43.17
EUR	LU - Luxembourg	594130	CIA	Jul-17	1	NR	NR	4	6	11	ViewHub	2015	Unknown
EUR	LV - Latvia	1944643	CIA	Jul-17	3	1	1	108	167	280	ViewHub	2015	14.40
EUR	MC - Monaco	30645	CIA	Jul-17	NR	NR	NR	2	3	5	ViewHub	2015	Unknown
EUR	MD - Republic of Moldova	3474121	CIA	Jul-17	16	6	5	493	761	1281	ViewHub	2015	36.87
EUR	ME - Montenegro	642550	CIA	Jul-17	2	1	1	54	83	141	ViewHub	2015	21.94
EUR	MK - Macedonia	2103721	CIA	Jul-17	19	7	6	193	261	486	ViewHub	2015	23.10
EUR	MT - Malta	416338	CIA	Jul-17	9	3	3	14	21	50	ViewHub	2015	12.01
EUR	NL - Netherlands	17084719	CIA	Jul-17	18	7	6	170	262	463	ViewHub	2015	2.71
EUR	NO - Norway	5320045	CIA	Jul-17	4	1	1	44	68	118	ViewHub	2015	2.22
EUR	PL - Poland	38476269	CIA	Jul-17	137	52	46	2925	4511	7671	ViewHub	2015	19.94
EUR	PT - Portugal	10839514	CIA	Jul-17	7	3	2	97	149	258	ViewHub	2015	2.38
EUR	RO - Romania	21529967	CIA	Jul-17	190	71	63	1355	2090	3769	ViewHub	2015	17.51
EUR	RS - Serbia	7111024	CIA	Jul-17	14	5	5	661	1020	1705	ViewHub	2015	23.98
EUR	RU - Russian Federation	142257519	CIA	Jul-17	1704	641	564	26879	41461	71249	ViewHub	2015	50.08
EUR	SE - Sweden	9960487	CIA	Jul-17	5	2	2	149	229	387	ViewHub	2015	3.89
EUR	SI - Slovenia	1972126	CIA	Jul-17	NR	NR	NR	71	109	180	ViewHub	2015	Unknown
EUR	SK - Slovakia	5445829	CIA	Jul-17	15	5	5	143	221	389	ViewHub	2015	7.14
EUR	SM - San Marino	33537	CIA	Jul-17	NR	NR	NR	1	1	2	ViewHub	2015	Unknown
EUR	TR - Turkey	80845215	CIA	Jul-17	1032	388	342	4461	6881	13104	ViewHub	2015	16.21
EUR	UA - Ukraine	44033874	CIA	Jul-17	191	72	63	7217	11132	18675	ViewHub	2015	42.41
EUR	UK - United Kingdom	64769452	CIA	Jul-17	83	31	27	488	753	1382	ViewHub	2015	2.13
EUR	UZ - Uzbekistan	29748859	CIA	Jul-17	1193	449	395	4685	7226	13948	ViewHub	2015	46.89
EUR	XK - Kosovo	1895250	CIA	Jul-17	N/A	N/A	N/A	N/A	N/A	N/A	N/A	N/A	Unknown
SEAR	BD - Bangladesh	157826578	CIA	Jul-17	2481	933	822	101842	259285	365363	ViewHub	2015	231.50
SEAR	BT - Bhutan	758288	CIA	Jul-17	17	6	6	325	834	1188	ViewHub	2015	156.67
SEAR	ID - Indonesia	260580739	CIA	Jul-17	7498	2820	2483	230600	585770	829171	ViewHub	2015	318.20
SEAR	IN - India	1281935911	CIA	Jul-17	64538	24133	21373	1229970	3133014	4473028	ViewHub	2015	348.93
SEAR	KP - North Korea	25248140	CIA	Jul-17	660	248	219	7172	18438	26737	ViewHub	2015	105.90
SEAR	KR - South Korea	51181299	CIA	Jul-17	239	90	79	6002	15398	21808	ViewHub	2015	42.61
SEAR	LK - Sri Lanka	22409381	CIA	Jul-17	96	36	32	8987	22742	31893	ViewHub	2015	142.32
SEAR	MM - Myanmar	55123814	CIA	Jul-17	2423	911	802	36962	94384	135482	ViewHub	2015	245.78
SEAR	MV - Maldives	392709	CIA	Jul-17	1	1	1	178	453	634	ViewHub	2015	161.44
SEAR	MY - Malaysia	31381992	CIA	Jul-17	638	240	211	11070	28350	40509	ViewHub	2015	129.08
SEAR	NP - Nepal	29384297	CIA	Jul-17	553	208	183	18419	46732	66095	ViewHub	2015	224.93
SEAR	TH - Thailand	68414135	CIA	Jul-17	313	118	104	17853	45873	64261	ViewHub	2015	93.93
SEAR	TL - Timor Leste	1291358	CIA	Jul-17	141	53	47	1524	3942	5707	ViewHub	2015	441.94
WPR	AS - American Samoa	51504	CIA	Jul-17	N/A	N/A	N/A	N/A	N/A	N/A	N/A	N/A	Unknown
WPR	AU - Australia	23232413	CIA	Jul-17	36	14	12	498	769	1329	ViewHub	2015	5.72
WPR	BN - Brunei Darussalam	443593	CIA	Jul-17	3	1	1	50	76	131	ViewHub	2015	29.53
WPR	CK - Cook Islands	9290	CIA	Jul-17	1	NR	NR	17	43	61	ViewHub	2015	Unknown
WPR	CN - China	1379302771	CIA	Jul-17	26434	9943	8754	196288	506501	747920	ViewHub	2015	54.22
WPR	FJ - Fiji	920938	CIA	Jul-17	49	19	16	184	472	740	ViewHub	2015	80.35

WPR	FM - Micronesia	104196	CIA	Jul-17	1	1	1	36	92	131	ViewHub	2015	125.72
WPR	GU - Guam	167358	CIA	Jul-17	N/A	N/A	N/A	N/A	N/A	N/A	N/A	N/A	Unknown
WPR	HK - Hong Kong	7191503	CIA	Jul-17	N/A	N/A	N/A	N/A	N/A	N/A	N/A	N/A	Unknown
WPR	JP - Japan	126451398	CIA	Jul-17	138	52	46	4353	6714	11303	ViewHub	2015	8.94
WPR	KH - Cambodia	16204486	CIA	Jul-17	359	135	119	9860	25084	35557	ViewHub	2015	219.43
WPR	KI - Kiribati	108145	CIA	Jul-17	3	1	1	43	109	157	ViewHub	2015	145.18
WPR	LA - Lao People's Democratic Republic	7126706	CIA	Jul-17	280	105	93	5709	14542	20729	ViewHub	2015	290.86
WPR	MH - Marshall Islands	74539	CIA	Jul-17	1	NR	NR	21	53	75	ViewHub	2015	Unknown
WPR	MN - Mongolia	3068243	CIA	Jul-17	315	119	104	1245	3202	4985	ViewHub	2015	162.47
WPR	MO - Macao	601969	CIA	Jul-17	N/A	N/A	N/A	N/A	N/A	N/A	N/A	N/A	Unknown
WPR	MP - Northern Mariana Islands	52263	CIA	Jul-17	N/A	N/A	N/A	N/A	N/A	N/A	N/A	N/A	Unknown
WPR	NC - New Caledonia	279070	CIA	Jul-17	N/A	N/A	N/A	N/A	N/A	N/A	N/A	N/A	Unknown
WPR	NR - Nauru	9642	CIA	Jul-17	NR	NR	NR	4	10	14	ViewHub	2015	Unknown
WPR	NU - Niue	1626	CIA	Jun-15	NR	NR	NR	NR	NR	NR	ViewHub	2015	NR
WPR	NZ - New Zealand	4510327	CIA	Jul-17	28	10	9	118	181	346	ViewHub	2015	7.67
WPR	PF - French Polynesia	287881	CIA	Jul-17	N/A	N/A	N/A	N/A	N/A	N/A	N/A	N/A	Unknown
WPR	PG - Papua New Guinea	6909701	CIA	Jul-17	319	120	106	5778	14709	21032	ViewHub	2015	304.38
WPR	PH - Philippines	104256076	CIA	Jul-17	2760	1038	914	83555	211269	299536	ViewHub	2015	287.31
WPR	PN - Pitcairn Islands	54	CIA	Jul-16	N/A	N/A	N/A	N/A	N/A	N/A	N/A	N/A	Unknown
WPR	PW - Palau	21431	CIA	Jul-17	1	NR	NR	3	6	10	ViewHub	2015	Unknown
WPR	SB - Solomon Islands	647581	CIA	Jul-17	15	5	5	393	1006	1424	ViewHub	2015	219.90
WPR	SG - Singapore	5888926	CIA	Jul-17	8	3	3	258	398	670	ViewHub	2015	11.38
WPR	TK - Tokelau	1285	CIA	2016	N/A	N/A	N/A	N/A	N/A	N/A	N/A	N/A	Unknown
WPR	TO - Tonga	106479	CIA	Jul-17	9	3	3	54	139	208	ViewHub	2015	195.34
WPR	TV - Tuvalu	11052	CIA	Jul-17	1	1	NR	4	10	16	ViewHub	2015	Unknown
WPR	TW - Taiwan	23508428	CIA	Jul-17	N/A	N/A	N/A	N/A	N/A	N/A	N/A	N/A	Unknown
WPR	VN - Vietnam	96160163	CIA	Jul-17	2795	1051	926	52475	134841	192088	ViewHub	2015	199.76
WPR	VU - Vanuatu	282814	CIA	Jul-17	7	3	2	228	584	824	ViewHub	2015	291.36
WPR	WF - Wallis and Futuna	15714	CIA	Jul-17	N/A	N/A	N/A	N/A	N/A	N/A	N/A	N/A	Unknown
WPR	WS - Samoa	200108	CIA	Jul-17	11	4	4	103	267	389	ViewHub	2015	194.40

N/A - not available
NR - not reported
Unknown - not enough data available
CIA - Central Intelligence Agency (World Factbook 2018)
ViewHub uses WHO surveillance data
