# Supplementary material for: Identifying UK travellers at increased risk of developing pneumococcal infection: a novel algorithm
Source: J Travel Med. 2021 May 12;28(6):taab063. doi: 10.1093/jtm/taab063 (PMC8393689; doi:10.1093/jtm/taab063)
Supplement: Supplementary_table_Vaccine_start_date_200630_taab063 [file supplementary_table_vaccine_start_date_200630_taab063.pdf]

| **Region** (WHO regional codes) | **Country** (2 letter country codes - country name) | **Vaccine introduction date (month/day/year)** | **Data source** | **Duration of PCV programme** (number of completed years up to 01 May 2020) |
|---|---|---|---|---|
| AFR | AO - Angola | 06/03/2013 | ViewHub | 6 |
| AFR | BF - Burkina Faso | 10/31/2013 | ViewHub | 6 |
| AFR | BI - Burundi | 09/20/2011 | ViewHub | 8 |
| AFR | BJ - Benin | 07/26/2011 | ViewHub | 8 |
| AFR | BW - Botswana | 07/03/2012 | ViewHub | 7 |
| AFR | CD - Democratic Republic of the Congo | 01/01/2012 | ViewHub | 8 |
| AFR | CF - Central African Republic | 06/30/2011 | ViewHub | 8 |
| AFR | CG - Congo | 10/11/2012 | ViewHub | 7 |
| AFR | CI - Côte d'Ivoire | 09/30/2014 | ViewHub | 5 |
| AFR | CM - Cameroon | 07/01/2011 | ViewHub | 8 |
| AFR | Comoros | Start 2021 | WHO | 0 |
| AFR | CV - Cape Verde | N/A | ViewHub/WHO | No programme |
| AFR | DZ - Algeria | 2016 | WHO | 3 |
| AFR | ER - Eritrea | 08/01/2015 | ViewHub | 4 |
| AFR | ET - Ethiopia | 10/15/2011 | ViewHub | 8 |
| AFR | GA - Gabon | N/A | ViewHub/WHO | No programme |
| AFR | GH - Ghana | 04/26/2012 | ViewHub | 8 |
| AFR | GM - Gambia | 08/19/2009 | ViewHub | 10 |
| AFR | GN - Guinea | N/A | ViewHub/WHO | No programme |
| AFR | GQ - Equatorial Guinea | N/A | ViewHub/WHO | No programme |
| AFR | GW - Guinea-Bissau | 06/19/2015 | ViewHub | 4 |
| AFR | KE - Kenya | 02/14/2011 | ViewHub | 9 |
| AFR | LR - Liberia | 01/09/2014 | ViewHub | 6 |
| AFR | LS - Lesotho | 07/10/2015 | ViewHub | 4 |
| AFR | MG - Madagascar | 11/05/2012 | ViewHub | 7 |
| AFR | ML - Mali | 03/15/2011 | ViewHub | 9 |
| AFR | MR - Mauritania | 11/12/2013 | ViewHub | 6 |

| | | | | |
|---|---|---|---|---|
| AFR | MU - Mauritius | 03/24/2016 | ViewHub | 4 |
| AFR | MW - Malawi | 11/12/2011 | ViewHub | 8 |
| AFR | MZ - Mozambique | 04/01/2013 | ViewHub | 7 |
| AFR | NA - Namibia | 11/11/2014 | ViewHub | 5 |
| AFR | NE - Niger | 08/05/2014 | ViewHub | 5 |
| AFR | NG - Nigeria | 12/22/2014 | ViewHub | 5 |
| AFR | RE - Reunion | NR | ViewHub/WHO | Unknown |
| AFR | RW - Rwanda | 07/13/2009 | ViewHub | 10 |
| AFR | SC - Seychelles | 09/01/2018 | ViewHub | 1 |
| AFR | SL - Sierra Leone | 01/28/2011 | ViewHub | 9 |
| AFR | SN - Senegal | 11/05/2013 | ViewHub | 6 |
| AFR | SO - Somalia | N/A | ViewHub/WHO | No programme |
| AFR | South Sudan | N/A | ViewHub/WHO | No programme |
| AFR | ST - Sao Tome and Principe | 11/30/2012 | ViewHub | 7 |
| AFR | SZ - Swaziland | 04/23/2014 | ViewHub | 6 |
| AFR | TD - Chad | N/A | ViewHub/WHO | No programme |
| AFR | TG - Togo | 06/19/2014 | ViewHub | 5 |
| AFR | TZ - United Republic of Tanzania | 12/06/2012 | ViewHub | 7 |
| AFR | UG - Uganda | 04/27/2013 | ViewHub | 7 |
| AFR | Western Sahara | NR | ViewHub/WHO | Unknown |
| AFR | ZA - South Africa | 04/01/2009 | ViewHub | 11 |
| AFR | ZM - Zambia | 05/10/2013 | ViewHub | 6 |
| AFR | ZW - Zimbabwe | 07/26/2012 | ViewHub | 7 |
| AMR | AG - Antigua and Barbuda | N/A | ViewHub/WHO | Risk groups |
| AMR | AI - Anguilla | NR | ViewHub/WHO | Unknown |
| AMR | AR - Argentina | 01/01/2012 | ViewHub | 8 |
| AMR | AW - Aruba | NR | ViewHub/WHO | Unknown |
| AMR | BB - Barbados | 01/01/2009 | ViewHub | 11 |
| AMR | BM - Bermuda | NR | ViewHub/WHO | Unknown |
| AMR | BO - Bolivia | 01/30/2014 | ViewHub | 6 |
| AMR | BR - Brazil | 11/01/2010 | ViewHub | 9 |
| AMR | BS - Bahamas | 01/01/2009 | ViewHub | 11 |
| AMR | BZ - Belize | N/A | ViewHub/WHO | No programme |

| | | | | |
|---|---|---|---|---|
| AMR | CA - Canada | 01/01/2002 | ViewHub | 18 |
| AMR | Caribbean Netherlands | NR | ViewHub/WHO | Unknown |
| AMR | CL - Chile | 01/01/2011 | ViewHub | 9 |
| AMR | CO - Columbia | 09/01/2011 | ViewHub | 8 |
| AMR | CR - Costa Rica | 01/01/2008 | ViewHub | 12 |
| AMR | CU - Cuba | N/A | ViewHub/WHO | No programme |
| AMR | CW - Curaçao | NR | ViewHub/WHO | Unknown |
| AMR | DM - Dominica | N/A | ViewHub/WHO | No programme |
| AMR | DO - Dominican Republic | 07/01/2013 | ViewHub | 6 |
| AMR | EC - Ecuador | 08/01/2010 | ViewHub | 9 |
| AMR | FK - Falkland Islands | NR | ViewHub/WHO | Unknown |
| AMR | GD - Grenada | N/A | ViewHub/WHO | No programme |
| AMR | GF - French Guiana | NR | ViewHub/WHO | Unknown |
| AMR | GP - Guadeloupe | NR | ViewHub/WHO | Unknown |
| AMR | Greenland | NR | ViewHub/WHO | Unknown |
| AMR | GT - Guatemala | 01/01/2012 | ViewHub | 8 |
| AMR | GY - Guyana | 01/10/2011 | ViewHub | 9 |
| AMR | HN - Honduras | 04/01/2011 | ViewHub | 9 |
| AMR | HT - Haiti | 11/2018 | ViewHub/WHO | 1 |
| AMR | JM - Jamaica | 07/01/2010 | ViewHub | 9 (Risk groups) |
| AMR | KN - Saint Kitts and Nevis | N/A | ViewHub/WHO | No programme |
| AMR | KY - Cayman Islands | N/A | ViewHub/WHO | Unknown |
| AMR | LC - Saint Lucia | N/A | ViewHub/WHO | Infants at risk |
| AMR | Montserrat | NR | ViewHub/WHO | Unknown |
| AMR | MQ - Martinique | NR | ViewHub/WHO | Unknown |
| AMR | MX - Mexico | 03/01/2008 | ViewHub | 12 |
| AMR | NI - Nicaragua | 12/12/2010 | ViewHub | 9 |
| AMR | PA - Panama | 01/01/2010 | ViewHub | 10 |
| AMR | PE - Peru | 01/01/2009 | ViewHub | 11 |
| AMR | PR - Puerto Rico | NR | ViewHub/WHO | Unknown |
| AMR | PY - Paraguay | 03/01/2012 | ViewHub | 8 |
| AMR | SR - Suriname | N/A | ViewHub/WHO | No programme |
| AMR | SV - El Salvador | 11/01/2010 | ViewHub | 9 |

| | | | | |
|---|---|---|---|---|
| AMR | SX - Sint Maarten | NR | ViewHub/WHO | Unknown |
| AMR | TC - Turks and Caicos | NR | ViewHub/WHO | Unknown |
| AMR | TT - Trinidad and Tobago | 01/01/2009 | ViewHub | 11 |
| AMR | United States Virgin Islands | NR | ViewHub/WHO | Unknown |
| AMR | US - United States of America | 07/01/2000 | ViewHub | 19 |
| AMR | UY - Uruguay | 03/01/2008 | ViewHub | 12 |
| AMR | VC - Saint Vincent and the Grenadines | N/A | ViewHub/WHO | No programme |
| AMR | VE - Venezuela | 07/01/2014 | ViewHub | 5 |
| AMR | VG - British Virgin Islands | NR | ViewHub/WHO | Unknown |
| EUR | AD - Andorra | 01/01/2007 | ViewHub | 13 |
| EUR | AL - Albania | 03/12/2011 | ViewHub | 9 |
| EUR | AM - Armenia | 09/15/2014 | ViewHub | 5 |
| EUR | AT - Austria | 01/01/2002 | ViewHub | 18 |
| EUR | AZ - Azerbaijan | 12/01/2013 | ViewHub | 6 |
| EUR | BA - Bosnia and Herzegovina | N/A | ViewHub/WHO | Risk groups |
| EUR | BE - Belgium | 01/01/2006 | ViewHub | 14 |
| EUR | BG - Bulgaria | 06/01/2010 | ViewHub | 9 |
| EUR | BY - Belarus | 01/01/2014 | ViewHub | 6 (Risk groups) |
| EUR | CH - Switzerland | 01/01/2006 | ViewHub | 14 |
| EUR | CY - Cyprus | 01/01/2007 | ViewHub | 13 |
| EUR | CZ - Czech Republic | 01/01/2010 | ViewHub | 10 |
| EUR | DE - Germany | 07/01/2006 | ViewHub | 13 |
| EUR | DK - Denmark | 10/01/2007 | ViewHub | 12 |
| EUR | EE - Estonia | 07/01/2014 | ViewHub | 5 (Risk groups) |
| EUR | ES - Spain | 06/01/2001 (Regional programme) | ViewHub | 18 |
| EUR | FI - Finland | 09/01/2010 | ViewHub | 9 |
| EUR | FO - Faroe Islands | NR | ViewHub/WHO | Unknown |
| EUR | FR - France | 05/01/2006 | ViewHub | 14 |
| EUR | GE - Georgia | 11/24/2014 | ViewHub | 5 |
| EUR | GI - Gibraltar | NR | ViewHub/WHO | Unknown |
| EUR | GR - Greece | 01/01/2006 | ViewHub | 14 |

| | | | | |
|---|---|---|---|---|
| EUR | HR - Croatia | N/A | ViewHub/WHO | Unknown |
| EUR | HU - Hungary | 04/01/2009 | ViewHub | 11 |
| EUR | IE - Ireland | 09/01/2008 | ViewHub | 11 |
| EUR | IL - Israel | 07/01/2009 | ViewHub | 10 |
| EUR | IS - Iceland | 04/01/2011 | ViewHub | 9 |
| EUR | IT - Italy | 05/01/2005 | ViewHub | 15 |
| EUR | KG - Kyrgyzstan | 03/21/2016 | ViewHub | 4 |
| EUR | KZ - Kazakhstan | 01/01/2011 | ViewHub | 9 |
| EUR | LI - Liechtenstein | NR | ViewHub/WHO | Unknown |
| EUR | LT - Lithuania | 10/01/2014 | ViewHub | 5 |
| EUR | LU - Luxembourg | 01/01/2005 | ViewHub | 15 |
| EUR | LV - Latvia | 01/01/2010 | ViewHub | 10 |
| EUR | MC - Monaco | 01/01/2006 | ViewHub | 14 |
| EUR | MD - Republic of Moldova | 10/01/2013 | ViewHub | 6 |
| EUR | ME - Montenegro | N/A | ViewHub/WHO | No programme |
| EUR | MK - Macedonia | N/A | ViewHub/WHO | Unknown |
| EUR | MT - Malta | Planned 10/2019 | WHO | <1 |
| EUR | NL - Netherlands | 06/01/2006 | ViewHub | 13 |
| EUR | NO - Norway | 07/01/2006 | ViewHub | 13 |
| EUR | PL - Poland | 03/01/2006 | ViewHub | 14 |
| EUR | PT - Portugal | 07/01/2015 | ViewHub | 4 |
| EUR | RO - Romania | N/A | ViewHub | Unknown |
| EUR | RS - Serbia | 04/01/2019 | WHO | 1 |
| EUR | RU - Russian Federation | 03/01/2014 | ViewHub | 6 |
| EUR | SE - Sweden | 01/01/2009 | ViewHub | 11 |
| EUR | SI - Slovenia | 01/01/2005 | ViewHub | 15 |
| EUR | SK - Slovakia | 01/01/2009 | ViewHub | 11 |
| EUR | SM - San Marino | N/A | ViewHub/WHO | Unknown |
| EUR | TR - Turkey | 11/01/2008 | ViewHub | 11 |
| EUR | UA - Ukraine | N/A | ViewHub/WHO | No programme |
| EUR | UK - United Kingdom | 09/01/2006 | ViewHub | 13 |
| EUR | UZ - Uzbekistan | 11/02/2015 | ViewHub | 4 |
| EUR | XK - Kosovo | NR | ViewHub | Unknown |

| | | | | |
|---|---|---|---|---|
| EMR | AE - United Arab Emirates | 06/01/2007 | ViewHub | 12 |
| EMR | AF - Afghanistan | 12/07/2013 | ViewHub | 6 |
| EMR | BH - Bahrain | 06/01/2008 | ViewHub | 11 |
| EMR | DJ - Djibouti | 12/06/2012 | ViewHub | 7 |
| EMR | EG - Egypt | N/A | ViewHub/WHO | No programme |
| EMR | IQ - Iraq | 03/01/2017 | ViewHub | 3 |
| EMR | IR - Iran (Islamic Republic of) | N/A | ViewHub/WHO | Risk groups |
| EMR | JO - Jordan | N/A | ViewHub | No programme |
| EMR | KW - Kuwait | 01/01/2007 | ViewHub | 13 |
| EMR | LB - Lebanon | 06/01/2015 | ViewHub | 4 |
| EMR | LY - Libya | 10/01/2013 | ViewHub | 6 |
| EMR | MA - Morocco | 10/20/2010 | ViewHub | 9 |
| EMR | OM - Oman | 01/01/2008 | ViewHub | 12 |
| EMR | PK - Pakistan | 10/09/2012 | ViewHub | 7 |
| EMR | QA - Qatar | 01/01/2005 | ViewHub | 15 |
| EMR | SA - Saudi Arabia | 03/01/2009 | ViewHub | 11 |
| EMR | SD - Sudan | 08/01/2013 | ViewHub | 6 |
| EMR | SY - Syria | N/A | ViewHub/WHO | No programme |
| EMR | TJ - Tajikistan | N/A | ViewHub/WHO | No programme |
| EMR | TM - Turkmenistan | Planned 10/2019 | WHO | <1 |
| EMR | TN - Tunisia | 01/04/2019 | ViewHub | 1 |
| EMR | YE - Yemen | 01/29/2011 | ViewHub | 9 |
| SEAR | BD - Bangladesh | 03/21/2015 | ViewHub | 5 |
| SEAR | BT - Bhutan | N/A | ViewHub/WHO | No programme |
| SEAR | ID - Indonesia | 03/22/2018 (Phased) | ViewHub | 2 |
| SEAR | IN - India | 05/13/2017 (Phased) | ViewHub | 2 |
| SEAR | KP - North Korea | NR | ViewHub/WHO | Unknown |
| SEAR | KR - South Korea | 05/01/2014 | ViewHub | 6 (Rep of Korea) |
| SEAR | LK - Sri Lanka | N/A | ViewHub/WHO | No programme |
| SEAR | MM - Myanmar | 07/01/2016 | ViewHub | 3 |
| SEAR | MV - Maldives | N/A | ViewHub/WHO | No programme |

| | | | | |
|---|---|---|---|---|
| SEAR | MY - Malaysia | N/A | ViewHub/WHO | No programme |
| SEAR | NP - Nepal | 01/19/2015 | ViewHub | 5 |
| SEAR | TH - Thailand | N/A | ViewHub/WHO | No programme |
| SEAR | TL - Timor Leste | N/A | ViewHub/WHO | No programme |
| WPR | AS - American Samoa | NR | ViewHub/WHO | No programme |
| WPR | AU - Australia | 01/01/2005 | ViewHub | 15 |
| WPR | BN - Brunei Darussalam | N/A | ViewHub/WHO | Infants at risk |
| WPR | CK - Cook Islands | N/A | ViewHub/WHO | No programme |
| WPR | CN - China | N/A | ViewHub/WHO | No programme |
| WPR | FJ - Fiji | 10/29/2012 | ViewHub | 7 |
| WPR | FM - Micronesia | 03/01/2008 | ViewHub | 12 |
| WPR | GU - Guam | NR | ViewHub/WHO | Unknown |
| WPR | HK - Hong Kong | 10/2010 | HK Government | 9 |
| WPR | JP - Japan | 01/01/2011 | ViewHub | 9 |
| WPR | KH - Cambodia | 01/14/2015 | ViewHub | 5 |
| WPR | KI - Kiribati | 04/01/2013 | ViewHub | 7 |
| WPR | LA - Lao People's Democratic Republic | 10/02/2013 | ViewHub | 6 |
| WPR | MH - Marshall Islands | 01/01/2009 | ViewHub | 11 |
| WPR | MN - Mongolia | 06/06/2016 (Phased) | ViewHub | 3 |
| WPR | MO - Macao | 09/2009 | Lee 2016 | 10 |
| WPR | MP - Northern Mariana Islands | NR | ViewHub/WHO | Unknown |
| WPR | NC - New Caledonia | NR | ViewHub/WHO | Unknown |
| WPR | NR - Nauru | N/A | ViewHub/WHO | No programme |
| WPR | NU - Niue | 01/01/2009 | ViewHub | 11 |
| WPR | NZ - New Zealand | 06/01/2008 | ViewHub | 11 |
| WPR | PF - French Polynesia | NR | ViewHub/WHO | Unknown |
| WPR | PG - Papua New Guinea | 11/12/2013 | ViewHub | 6 |
| WPR | PH - Philippines | 07/17/2013 (Phased) | ViewHub | 6 |
| WPR | PN - Pitcairn Islands | NR | ViewHub/WHO | Unknown |
| WPR | PW - Palau | 03/01/2008 | ViewHub | 12 |
| WPR | SB - Solomon Islands | 02/17/2015 | ViewHub | 5 |

| | | | | |
|---|---|---|---|---|
| WPR | SG - Singapore | 11/01/2009 | ViewHub | 10 |
| WPR | TK - Tokelau | NR | ViewHub/WHO | Unknown |
| WPR | TO - Tonga | 05/2019 | WHO | 1 |
| WPR | TV - Tuvalu | N/A | ViewHub/WHO | No programme |
| WPR | TW - Taiwan | 01/2015 | CDC | 5 |
| WPR | VN - Vietnam | N/A | ViewHub/WHO | No programme |
| WPR | VU - Vanuatu | N/A | ViewHub/WHO | No programme |
| WPR | WF - Wallis and Futuna | NR | ViewHub/WHO | Unknown |
| WPR | WS - Samoa | N/A | ViewHub/WHO | No programme |

N/A, not available; NR, not reported

ViewHub database - last updated 10 July 2018 (accessed June 2020)

WHO vaccine-preventable diseases: monitoring sysytem. 2019 global summary

HK government: https://www.chp.gov.hk/en/features/21730.html (accessed June 2020)

CDC: https://www.cdc.gov.tw/En/Category/ListContent/bg0g_VU_Ysrgkes_KRUDgQ?uaid=lS42udX_s0u2fN0qLcdrnw (accessed June 2020)
